# Systematic identification of factors involved in the silencing of germline genes in mouse embryonic stem cells

**DOI:** 10.1093/nar/gkad071

**Published:** 2023-02-11

**Authors:** Hala Al Adhami, Judith Vallet, Celia Schaal, Paul Schumacher, Anaïs Flore Bardet, Michael Dumas, Johana Chicher, Philippe Hammann, Sylvain Daujat, Michael Weber

**Affiliations:** University of Strasbourg, Strasbourg, France; CNRS UMR7242, Biotechnology and Cell Signaling, 300 Bd Sébastien Brant, 67412, Illkirch Cedex, France; University of Strasbourg, Strasbourg, France; CNRS UMR7242, Biotechnology and Cell Signaling, 300 Bd Sébastien Brant, 67412, Illkirch Cedex, France; University of Strasbourg, Strasbourg, France; CNRS UMR7242, Biotechnology and Cell Signaling, 300 Bd Sébastien Brant, 67412, Illkirch Cedex, France; University of Strasbourg, Strasbourg, France; CNRS UMR7242, Biotechnology and Cell Signaling, 300 Bd Sébastien Brant, 67412, Illkirch Cedex, France; Karlsruhe Institute of Technology (KIT), IAB, Department of Food Chemistry and Toxicology, 76131 Karlsruhe, Germany; University of Strasbourg, Strasbourg, France; CNRS UMR7242, Biotechnology and Cell Signaling, 300 Bd Sébastien Brant, 67412, Illkirch Cedex, France; University of Strasbourg, Strasbourg, France; CNRS UMR7242, Biotechnology and Cell Signaling, 300 Bd Sébastien Brant, 67412, Illkirch Cedex, France; Plateforme protéomique Strasbourg Esplanade, CNRS, University of Strasbourg, 67000 Strasbourg, France; Plateforme protéomique Strasbourg Esplanade, CNRS, University of Strasbourg, 67000 Strasbourg, France; University of Strasbourg, Strasbourg, France; CNRS UMR7242, Biotechnology and Cell Signaling, 300 Bd Sébastien Brant, 67412, Illkirch Cedex, France; University of Strasbourg, Strasbourg, France; CNRS UMR7242, Biotechnology and Cell Signaling, 300 Bd Sébastien Brant, 67412, Illkirch Cedex, France

## Abstract

In mammals, many germline genes are epigenetically repressed to prevent their illegitimate expression in somatic cells. To advance our understanding of the mechanisms restricting the expression of germline genes, we analyzed their chromatin signature and performed a CRISPR-Cas9 knock-out screen for genes involved in germline gene repression using a *Dazl*-GFP reporter system in mouse embryonic stem cells (mESCs). We show that the repression of germline genes mainly depends on the polycomb complex PRC1.6 and DNA methylation, which function additively in mESCs. Furthermore, we validated novel genes involved in the repression of germline genes and characterized three of them: *Usp7*, *Shfm1* (also known as *Sem1*) and *Erh*. Inactivation of *Usp7*, *Shfm1* or *Erh* led to the upregulation of germline genes, as well as retrotransposons for *Shfm1*, in mESCs. Mechanistically, USP7 interacts with PRC1.6 components, promotes PRC1.6 stability and presence at germline genes, and facilitates DNA methylation deposition at germline gene promoters for long term repression. Our study provides a global view of the mechanisms and novel factors required for silencing germline genes in embryonic stem cells.

## INTRODUCTION

The expression of many germline genes is normally suppressed in somatic lineages and restricted to germ cells, where they contribute to meiosis and gamete differentiation. Their expression needs to be tightly controlled because improper silencing of germline genes in somatic cells contributes to neoplasm and is a hallmark of aggressive cancer (1). Furthermore, their premature activation in primordial germ cells leads to precocious germline differentiation and impaired gametogenesis (2).

In mammals, the mechanisms of repression of germline genes are beginning to be elucidated and one of the main pathways is DNA methylation of CpG dinucleotides. In mice, a wave of demethylation occurs after fertilization followed by global *de novo* DNA methylation concomitant with implantation and epiblast formation (3,4). The establishment of DNA methylation is carried out by the *de novo* methyltransferases DNMT3A and DNMT3B while its maintenance requires DNMT1 and its cofactor UHRF1 (5). In contrast to interspersed CpG dinucleotides, most CpG islands (CGIs) remain protected against DNA methylation. During development, *de novo* methylation of CG-rich promoters is almost exclusively targeted to a subset of germline genes, leading to their long-term repression in somatic lineages (3,5). Indeed, these genes are de-repressed in DNA methylation deficient mouse embryos (5), including germline genes previously shown to be regulated by DNA methylation in murine embryonic fibroblasts (MEFs) (6). A second wave of demethylation occurs in primordial germ cells (PGCs) and many of these germline genes require DNA demethylation to be activated in PGCs (7).

In mESCs, other mechanisms cooperate with DNA methylation to repress germline genes. The histone methyltransferase SETDB1 responsible for H3K9me3 deposition is required for limiting the expression of several germline genes in mESCs (8,9). The combined inactivation of the H3K9me3 readers CBX1, CBX3 and CBX5 (also known as HP1b, HP1g and HP1a respectively) also leads to increased expression of germline genes (10). In addition, several studies revealed a central role for the non-canonical polycomb repressive complex PRC1.6 in the repression of germlines genes in embryonic stem cells. The PRC1.6 complex is composed of PCGF6, RYBP, L3MBTL2, CBX3, WDR5, the DNA binding subunits MAX, MGA, E2F6 and DP1, and the catalytic subunits RING1A/B (also known as RNF1/2) responsible for H2AK119ub deposition (11,12). The promoter sequences of many germline genes contain the E2F6 consensus sequence or an E-box motif recognized by MAX/MGA. Inactivation of E2F6, MAX or MGA reduces PRC1.6 recruitment to germline genes and reactivates germline genes in mESCs (13–17). Furthermore, inactivation of the other PRC1.6 components PCGF6, L3MBTL2, RYBP or the catalytic subunits RING1A/B also results in an up-regulation of overlapping sets of germline genes (12,15,18,19). Interestingly, E2F6, MGA and MAX repress germline genes in part by favoring H3K9me3 deposition at germline genes in mESCs (9,13,20).

Temporal analyses suggest that H3K9me3 and PRC1.6 play crucial roles at germline genes in naïve cells when DNA methylation is not yet established, whereas DNA methylation becomes the predominant mechanism in differentiated cells (13,17,20). Furthermore, DNA methylation of germline gene promoters is reduced in *Setdb1*, *Max, E2f6* or *L3mbtl2* knockout ESCs or embryos (9,13,21,22) and *Mga* mutant epiblast-like cells (20). These data suggest a stepwise mechanism by which SETDB1 and PRC1.6 repress germline genes before the global wave of DNA methylation, and subsequently favor DNA methylation of germline genes to establish long-term repression in post-implantation cells (13,20). Because ES cells cultured in standard serum and LIF conditions represent a mixed population of naïve and primed cells with distinct epigenetic states (23,24), they rely simultaneously on several mechanisms (such as H3K9me3, PRC1.6 and DNA methylation) to repress germline genes and represent a good model to study the multiple epigenetic mechanisms underlying germline genes repression during development.

Despite progress in the last years, the complete mechanisms involved in the silencing of germline genes in mammalian somatic cells remain unclear. To advance our understanding of germline gene regulation, we performed a computational analysis of their chromatin signature and performed a functional genome-wide CRISPR-Cas9 knock-out screen in mESCs. We uncover multiple factors involved in the repression of germline genes and present validation of three novel candidates. Our data provide a molecular roadmap of the mechanisms limiting the expression of germline genes in mouse embryonic stem cells and novel key factors involved in this process.

## MATERIALS AND METHODS

### Promoter annotation

We used RefSeq gene annotation and promoters were defined as −1000 bp to +500 bp around RefSeq TSS. For promoter classification based on CpG density, we calculated for each promoter the CpG ratio and GC content in 500 bp sliding windows with 20 bp increments. LCP were defined as containing no window with a CpG ratio >0.45, HCP were defined as containing at least one window with a CpG ratio >0.65 and a GC content >55%, and the remaining promoters were defined as ICP.

### Computational analysis of ChIP-seq data

Raw reads of ChIP-seq datasets for histone modifications, histone variants and proteins (Supplementary Table S1) were downloaded from GEO/SRA. The reads were trimmed using trim_galore (version 0.6.4 options -q 20 –stringency 2), aligned to the mouse genome (mm10) using bowtie2 (version 2.3.0) and selected if the mapping quality ≥10. Read density tracks were generated using genomeCoverageBed from bedtools, from reads extended to 200 bp and visualized using the IGV browser. For each dataset, we retrieved normalized read counts per base around each HCP TSS (−250 to +250 bp) with bwtool extract (version 1.0). We ranked the datasets based on the signal enrichment in gg-dko HCPs compared to all HCPs or inactive HCPs (defined as HCPs associated to genes with FPKM < 1 in WT ESCs).

### Cell culture

Mouse embryonic stem cell line E14TG2a was purchased from ATCC (CRL-1821, lot 62909865). J1 and Dnmt-TKO ES cells were a gift from M. Okano (25). ES cells were cultured in Glutamax and sodium pyruvate supplemented DMEM (Gibco) containing 15% Fetal Bovine Serum (FBS), 1000 U/ml LIF (Millipore), 0.1 mM non-essential amino acids, 50 U/ml penicillin and streptomycin and 0.1 mM 2-mercaptoethanol. Cells were adapted to gelatin without feeders after three passages. Immortalized MEFs were grown in DMEM supplemented with 10% FBS and 50 U/ml penicillin and streptomycin. All cells used in this study were tested negative for mycoplasma.

### Generation of GFP reporter ESC lines

sgRNAs targeting the *Dazl* exon 3 or *Mael* exon 3 were cloned in the pSpCas9(BB)-2A-Puro (PX459) plasmid (Addgene #62988) by BbsI digestion. Donor plasmids to insert GFP in the *Dazl* or *Mael* genes were generated by assembling four DNA fragments using the Gibson Assembly Master Mix (NEB #E2611S): a 5’ homology arm, a p2A-NLSX2-sfGFP cassette amplified by PCR from plasmid addgene #63709, a 3’ homology arm, and a modified pUC19 backbone vector (addgene #63709). 5’ and 3’ homology arms were amplified by PCR from genomic DNA extracted from E14 ESCs. All DNA fragments were gel purified before the Gibson assembly reaction. Donor plasmids were co-transfected with the PX459 vector in ESCs using lipofectamine 2000 (Invitrogen #10696153). Twenty-four hours after transfection, the cells were selected with 2 μg/ml puromycin (Thermo Fisher Scientific) for 48h, then plated at clonal density. Colonies were picked and expanded before checking the CRISPR-mediated insertion of the p2A-NLSx2-sfGFP sequence into the endogenous locus by PCR and sequencing.

### Preparation of the lentiviral sgRNA library for the CRISPR screen

The Brie lentiviral gRNA pooled library (in lentiGuide-Puro backbone, addgene #73633) was amplified by electroporating 400 ng of the pooled library into 100 μl electrocompetent bacteria (STBL4-TM, Invitrogen #11635-018). Electroporated bacteria were cultivated in 10 mL of SOC for 1 h at 30°C, then plated on 4 bioassay plates (500 cm^2^, LB agar + antibiotic) for 16 h at 30°C before plasmid purification with the NucleoBond Xtra Maxi Plus kit (Macherey-Nagel #740416.50). Lentiviruses were produced by transient co-transfection of 293T cells with a three-plasmid combination. 293T cells were plated at 15 million cells/15 cm dish the day before the transfection, and were transfected with 24 μg gRNA lentiviral vectors, 20.2 μg psPax2 packaging plasmid and 4.8 μg pVSV envelope plasmid using Polyethylenimine (PEI) transfection reagent (Tebu-bio #07923966-2). The culture supernatant was collected 48 and 72 h after transfection and concentrated using lentiX concentrator (TakaraBio #631231). The viral aliquots were kept at -80°C until usage. To calculate viral titers, 250 × 10³ ESCs per well of a 6-well plate were transduced with serial dilutions of viral concentrate in the presence of 4 μg/ml polybrene. Twenty-four hours later, cells were selected with 1 μg/ml puromycin for 7 days and the numbers of puromycin resistant colonies were counted using a crystal violet staining and a typhoon machine (GE Healthcare).

### CRISPR knockout screen

We first generated *Dazl*-GFP ESCs stably expressing Cas9 by transducing the *Dazl-*GFP clone with LentiCas9-blast lentiviruses (addgene #52962). Cells were selected with 10 μg/ml blasticidin for 10 days. To minimize clonal effects, the whole cellular pool was used to perform the screening. For the screening, we transduced 80 million *Dazl*-GFP ESCs expressing Cas9 with the Brie gRNA lentivirus library at a multiplicity of infection (MOI) of 0.3 using 4 μg/ml polybrene. Twenty-four hours later, the cells were selected with 1 μg/ml puromycin and 10 μg/ml blasticidin for 10 days with a medium change every 2 days. At day 10 post selection, 1/3 of cells were used as input and 2/3 were sorted by FACS to collect GFP+ cells using a FACSAria Fusion cell sorter (BD Biosciences). The screen was performed three times independently. Genomic DNA was extracted from sorted and input cells by phenol/chloroform extraction.

### CRISPR knockout screen sequencing and analysis

The whole amount of DNA from sorted cells (∼300–500 ng) was used for PCR, whereas 100 μg DNA was used from input cells to ensure a 300× coverage (20 PCR reactions on 5 μg each). PCR was performed with DreamTaq polymerase (Thermo Fisher Scientific) with a P5 primer and a unique P7 barcode primer with the following conditions: denaturation at 95°C for 3 min; 28 cycles of denaturation at 95°C for 30 s, annealing at 55°C for 30 s and extension at 72°C for 45 s; final extension at 72°C for 10 min. The PCR products were verified on an agarose gel and the libraries were then purified using Agencourt AmpureXP beads (Beckman-Coulter) and sequenced on an Illumina HiSeq 4000 sequencer to produce 75 bp paired-end reads by Integragen SA, France. The sgRNA distribution and enrichment were analyzed with MAGeCK-VISPR (26). For hit selection, we first removed olfactory receptor genes and selected the top 100 hits with a *P*-value <0.05. We then added the top 20 hits from each analysis of two replicates, and removed genes with a fpkm <5 in our RNA-seq analysis of E14 ESCs.

### Network analysis

Protein-protein interactions for the 76 selected candidates from the screen were performed using STRING v11.5. Interactions were computed using default parameters and network edges with a confidence score >0.4 were shown. Enrichment for mammalian phenotype was performed using the Enrichr gene set enrichment analysis tool (https://maayanlab.cloud/Enrichr).

### Gene ontology analysis

For each gene ontology biological process, we calculated the enrichment and associated hypergeometric *P*-values of genes in each class compared to all genes. *P*-values were then adjusted with Benjamini-Hochberg correction for multiple testing.

### Validation of candidates

For each candidate, one sgRNA from the Brie library was cloned in the Cas9-sgRNA lentiviral vector lentiCRISPRv2 (Addgene #52961) by BsmBI digestion. Lentiviral preparation was performed as described for the CRISPR library. ESCs (*Dazl-*GFP clone, *Mael-*GFP clone) were transduced, plated at 50 × 10³ cells/35 mm dish and 24 h later cells were selected with 1 μg/ml puromycin. GFP+ cells were counted after 6 or 8 days of selection using a FACSCalibur (BD Biosciences) and data were analyzed with the FlowJo software. gRNA sequences used to validate candidates are listed in the Supplementary Table S2.

### Generation of *Usp7* knockout ESCs lines

A gRNA targeting the exon 3 of the *Usp7* gene (GGTTGCCTCGGAGCGCCAAC) was cloned in the PX459 plasmid (Addgene #62988). Mouse ESCs E14TG2a were transfected with lipofectamine 2000 (Invitrogen #10696153) and selected for 48h by Puromycin (2 μg/ml). Single cells were sorted and plated at 1 cell/well in 96 well-plates. The clones were genotyped and tested for the presence of USP7 by Western blotting. We selected four independent *Usp7* KO clones and, as control, four independent E14TG2a WT clones. The mutations induced by CRISPR-Cas9 were confirmed by sanger sequencing.

### siRNA experiments

ON-TARGETplus SMART pool siRNAs (Horizon Discovery) were used to inhibit the expression of *Usp7*, *Dnmt1*, *Erh* or *Shfm1*. ON-TARGETplus Non-targeting Control siRNAs (Horizon Discovery) were used as negative control. ESCs cells were transfected with lipofectamine 2000 (Invitrogen #10696153) according to the manufacturer's instructions. 10 μl of lipofectamine 2000 diluted in 0.5 ml DMEM were mixed with 200 pmol siRNA in 0.5 ml DMEM and kept at room temperature for 20 min. The mix was added to a suspension of 1 ml ESCs (160 000 cells/ml) in ESC medium and plated on a 60 mm diameter petri dish. Medium was changed 6 h later and renewed every 24 h. 250 000 immortalized MEFs were transfected with 1000 pmol siRNA using the Neon electroporation system. Cells were harvested 48 or 72 h after siRNA transfection for protein and nucleic acid extraction. siRNA transfection experiments were performed in triplicates. The sequences of siRNAs are listed in the Supplementary Table S2.

### 5-Aza-dC treatment

5-Aza-2′-deoxycytidine (5-aza-dC) was purchased from Sigma (A3656) and prepared in water at 1 mg/ml stock concentration. ES cells or MEFs were treated with 0.5 μM final concentration of 5-aza-dC for 72 h with medium change every day.

### Nucleic acid extraction

DNA and RNA samples were extracted using the NucleoSpin RNA purification kit and NucleoSpin RNA/DNA Buffer Set for parallel RNA and DNA purification (Macherey-Nagel) according to the manufacturer's instructions and quantified using a Qubit 2.0 fluorometer (Life Technologies).

### RT-qPCR

RNAs were reverse transcribed with the Maxima first strand cDNA synthesis kit (Fisher Scientific #10282650) using a combination of oligo(dT) and random hexamer primers. RT-qPCR was performed with the KAPA SYBR FAST mix (Kapa Biosystems, KK4617) on a StepOnePlus realtime PCR system (Life Technologies) using the following PCR conditions: 95°C for 20 s, 40 cycles (95°C for 20 s, 64°C for 30 s), followed by a dissociation curve. The level of expression of each gene was calculated with the delta-delta Ct method and normalized with three housekeeping genes (*B2m, Gusb, Rpl13a*). The primer sequences are listed in Supplementary Table S2.

### Cobra

100 ng of genomic DNA was bisulfite-converted with the EpiTect bisulfite kit (Qiagen). The target region in the *Dazl* promoter was amplified by touchdown PCR with the Platinum Taq DNA Polymerase (Invitrogen) using the following conditions: 20 cycles of 30s at 95°C, 30s at 58–48°C (with a 0.5°C decrease per cycle), 50 s at 72°C followed by 35 cycles of 30 s at 95°C, 30 s at 48°C, 50 s at 72°C. The PCR products were purified using the PCR cleanup kit (Macherey-Nagel). 50 ng of PCR product were digested by TaqαI (Thermo Fisher Scientific) and loaded on an agarose gel alongside 50 ng of undigested PCR product. The primer sequences are listed in Supplementary Table S2.

### Transcriptome analysis by RNA-seq

RNA-seq was performed on RNAs from three independent siRNA experiments, as well as four independent WT and four independent *Usp7*-KO clones. Library preparation and sequencing was performed at the GenomEast platform. For siRNA experiments, RNA-seq libraries were prepared from 400 ng total RNA using the TruSeq Stranded Total RNA Library Prep Gold kit with Ribo-Zero rRNA removal and TruSeq RNA Single Indexes kits A and B (Illumina), according to the manufacturer's instructions. The final libraries were generated with 12 cycles of PCR amplification and purified using AMPure XP beads (Beckman-Coulter). For *Usp7* WT and KO clones, RNA-Seq libraries were generated from 100 ng total RNA using the Illumina Stranded Total RNA Prep with Ribo-Zero Plus kit and IDT for Illumina RNA UD Indexes (Illumina), according to the manufacturer's instructions. The final libraries were generated with 13 cycles of PCR amplification and purified using SPRIselect beads (Beckman-Coulter). RNA-seq libraries were checked for quality and quantified using capillary electrophoresis, and sequenced on an Illumina HiSeq 4000 by single-end (1 × 50 bp) sequencing. Reads were mapped to the mouse mm10 genome with TopHat v2.0.13 with a RefSeq transcriptome index. Reads were counted in RefSeq genes with HTseq-count v0.7.2 (parameters –t exon –s reverse) and differentially expressed genes were analyzed using DESeq2 v1.20.0. Genes were called differentially expressed if they had an adjusted *P*-value < 0.001 and a fold change >2 (siRNA experiments) or >3 (KO cells). For data visualization, bigwig files were generated using bam2wig.py from the RSeQC package v2.6.4 (parameters -u -t 5000000000) and visualized in the IGV browser. The FPKM values were calculated using DESeq2. The expression of transposable elements was analyzed by counting unique and multiple-mapping reads in RepeatMasker TE families using featureCounts from the Rsubread package (v1.30.9) with the option to weight multi-mapping reads by the number of mapping sites (parameters countMultiMappingReads = TRUE, fraction = TRUE, useMetaFeatures = TRUE). Differential expression of TE families was analyzed using DESeq2 v1.20.0. Volcano plots were generated using VolcaNoseR (https://huygens.science.uva.nl/VolcaNoseR). Germline genes were defined as genes showing a clear biased expression in testis in the BioGPS database.

### DNA methylome analysis by RRBS

RRBS libraries were prepared from 100 ng genomic DNA as described previously (27). Briefly, DNA was digested by MspI (Thermo Fisher Scientific), end-repaired and A-tailed with Klenow fragment exo- (Thermo Fisher Scientific) and ligated to methylated adapters with T4 DNA ligase (Thermo Fisher Scientific) in Tango 1X buffer. DNA fragments ranging from 150 to 400 bp were selected by gel excision, purified using the MinElute gel extraction kit (Qiagen) and bisulfite-converted twice with the EpiTect bisulfite kit (Qiagen). The libraries were amplified using the Pfu Turbo Cx hotstart DNA polymerase (Agilent) with 12 PCR cycles, purified using Agencourt AmpureXP beads (Beckman-Coulter) and sequenced (2 × 75 bp) on an Illumina HiSeq 4000 by Integragen SA, France. Reads were trimmed with Trim Galore (v0.4.4) in –non_directional and –rrbs mode to remove adapter sequences, two bases filled in during end-repair of MspI restriction sites, and low-quality ends with a Phred score below 20. Sequencing reads were mapped to the mouse genome (mm10) with Bismark v0.18.2 with default parameters. A maximum of two mismatches and an insertion size for paired-end sequences between 30 and 400 bp were allowed. Methylation scores were extracted as the ratio of the number of Cs over the total number of Cs and Ts using the Bismark_methylation_extractor. CpG methylation ratios from both strands were combined and filtered for a minimum sequencing depth of 8X. The bisulfite conversion efficiency was estimated by calculating the C to T conversion at the end-repaired MspI CpG sites, which was greater than 99% (Supplementary Table S3).

### Co-immunoprecipitation experiments

Total proteins were extracted in 50 mM Tris–HCI pH 7.5, 150 mM NaCl, 0.5 mM EDTA, 1% Triton X100 and 2× Protease inhibitors (Pierce #78442) and briefly sonicated. The endogenous USP7 was immunoprecipitated by overnight incubation at 4°C of 2 mg protein lysate with 6 μg anti-USP7 antibody (Bethyl A300-033A) followed by 1h30 incubation with Protein A/G magnetic beads (Pierce #88802) at 4°C. Protein complexes were washed 5 times with 50 mM Tris–HCI pH 7.5, 250 mM NaCl, 0.5 mM EDTA, 1% Triton X100, 2× protease inhibitors (Pierce #78442) and eluted in 2× SDS gel-loading buffer for 10 min at 95°C. *Usp7*-KO ES cells were used as negative controls for the immunoprecipitation experiments. For Western blot analysis, 15% of eluted proteins and 30 μg of input proteins were loaded on SDS-polyacrylamide gels.

### IP/MS analysis of USP7 partners

For mass spectrometry analyses, we performed three independent immunoprecipitation experiments as described above on WT and *Usp7*-KO ES cells. Eluted proteins were digested with sequencing-grade trypsin (Promega) and analysed by nanoLC–MS/MS on a QExactive Plus mass spectrometer coupled to an EASY-nanoLC-1000 (ThermoFisher Scientific). Peptides were identified with the Mascot algorithm (version 2.6, Matrix Science) against the Swissprot database with the *Mus musculus* taxonomy (release 2021_03) using the software's decoy strategy. Mascot identifications were imported into the Proline 2.0 software and validated using the following settings: Mascot pretty rank ≤1, FDR ≤1% for PSM scores, FDR ≤1% for protein set scores. The total number of MS/MS fragmentation spectra was used to quantify each protein. Statistical analysis of enriched proteins in WT compared to *Usp7*-KO cells was performed using R v4.0.3 and a homemade R package (IPinquiry4, https://github.com/) based on the msmsTests R package to process label-free LC-MS/MS data. The spectral counts were normalised using DESeq2 (median of ratios method) and EdgeR was used to perform a negative-binomial test and calculate the fold change and an adjusted *P*-value corrected by Benjamini–Hochberg for each protein. For this study, we defined significantly enriched proteins with a fold change >4 and an adjusted *P*-value <0.05.

### Chromatin immunoprecipitation

ESCs were fixed with 1% formaldehyde for 10 min at room temperature and quenched by 125 mM glycine for 5 min. Cells were scraped in 5 ml cold PBS on ice, rinsed once, and cell pellets corresponding to 10 × 10^6^ cells were snap-frozen in liquid nitrogen. Cell pellets were thawed on ice and lysed in 1 ml lysis buffer (50 mM HEPES–KOH pH 7.5, 140 mM NaCl, 1 mM EDTA, 10% glycerol, 0.5% NP40, 0.25% Triton X-100, 1× protease inhibitors) at 4°C for 10 min. The nuclei were washed in 10 mM Tris–HCl pH 8.0, 200 mM NaCl, 1 mM EDTA, 0.5 mM EGTA, 1× protease inhibitors (Pierce #78442), then resuspended in 500 μl shearing buffer (10 mM Tris pH 8.0, 100 mM NaCl, 1 mM EDTA, 0.5 mM EGTA, 0.5% *N*-lauroylsarcosine, 0.1% Na-deoxycholate, 2× protease inhibitors) and sonicated with a Covaris E220 sonicator. The sonicated lysates were centrifuged at 16 000g for 15 min at 4°C to pellet cellular debris and the supernatant was transferred to a new 1.5 ml LoBind Eppendorf tube. Chromatin was quantified using the Qubit dsDNA BR Assay Kit (Invitrogen #Q32853). 21 μg of chromatin were immunoprecipitated in 200 μl shearing buffer with 1% Triton X-100 overnight at 4°C with 2 μg anti-MGA antibody (Abcam #ab214814) or anti-PCGF6 antibody (Proteintech #24103-1-AP) or IgG control antibody (Millipore #CS200581) pre-incubated with 5 μl Blocker (Active Motif #37498). The immunocomplexes were collected on Protein G Agarose Columns (Active Motif #53039) for 3 h at 4°C, washed five times at 4°C with 900 μl wash Buffer (50 mM HEPES–KOH pH 7.6, 0.5 M LiCl, 1 mM EDTA, 0.7% sodium deoxycholate, 1% NP-40) followed by 2 washes with 900 μl TE-plus-50 mM NaCl (10 mM Tris–HCl pH 8.0, 50 mM NaCl, 1 mM EDTA), and eluted at 37°C for 5 min with Elution Buffer (50 mM Tris–HCl pH 8.0, 10 mM EDTA, 1% SDS). Input and ChIP samples were treated with 1 μl RNase A/T1 Mix (Thermo Scientific #EN0551) and reverse-crosslinked with 0.5% SDS and 130 ng/μl Proteinase K (Euromedex #EU0090-B) at 55°C for 1 h then at 65°C for 16 h. DNA was purified with the NucleoSpin Gel and PCR Clean up kit and Binding Buffer NTB (Macherey Nagel #740609). The enrichment of target sequences in the ChIP samples was determined by qPCR with the KAPA SYBR FAST mix (Kapa Biosystems #KK4617) on a StepOnePlus realtime PCR system (Applied Biosystem).

### Western blotting

Whole cell extracts were prepared by cell lysis in RIPA buffer (Clinisciences) for 30 min on ice followed by brief sonication. 30 μg of protein extracts were run on precast mini Protean SDS-polyacrylamide normal or gradient (4–20%) gels (BioRad) and transferred to 0.45 or 0.2 μm nitrocellulose membranes using the BioRad Trans-blot Turbo Transfer System. The membranes were blocked in blocking solution (TBS, 5% milk, 0.1% Tween-20) for 1 h at room temperature. The membranes were incubated at 4°C O/N with the primary antibodies diluted in blocking solution, and then for 1 h at room temperature with horseradish peroxidase-conjugated anti-mouse or -rabbit secondary antibodies (HealthcareDako P-0447 at 1/10 000 and Jackson Immunoresearch 111-035-003 at 1:100000) for ECL detection. For quantification of PRC1.6 proteins, the membranes were incubated with Alexa Fluor 680-conjugated secondary antibodies (Invitrogen A21057 and A21109 both at 1:20000) followed by fluorescence detection with a LI-COR Odyssey DLx imaging system and quantification with the LI-COR Empiria Studio software. For SHFM1 detection, 80 μg of protein extracts were run on a 16.5% Mini-PROTEAN Tris-Tricine Gel (BioRad #4563063) at 100 V for 4 h and transferred to a 0.2 μM PVDF Membrane (BioRad #1704156) using the TransBlot Turbo Transfer System (1.3 A, 25 V maximum, 4 min). After blocking (TBS, 5% milk, 0.05% tween-20) for 1 h at room temperature, the membrane was incubated at 4°C O/N with the primary antibody and the signal was revealed by ECL using the SuperSignal West Femto Maximum Sensitivity Substrate (Thermo Scientific #34094). The following primary antibodies were used in this study: USP7 (Bethyl #A300-033A, 1:15 000 in all figures except when indicated Abcam #ab109109, 1:500), ACTIN (Sigma #A2066, 1:4000), α-TUBULIN (Sigma #T9026, 1:10 000), LAMIN-B1 (Abcam #ab16048, 1:2000), VINCULIN (Abcam #ab129002, 1:10 000), DNMT1 (Cell Signaling Technology #5032, 1:1000), MGA (Abcam #ab214814, 1:667), PCGF6 (Abcam #ab200038, 1:1000), L3MBTL2 (Active Motif #39570, 1:500), E2F6 (Kerafast #LLF6-2, 1:500), MAX (Santa Cruz Biotechnology #sc-197, 1:200), RING1B (MBL #D139-3, 1:1000), ERH (Abcam #ab166620, 1:500), SHFM1 (Invitrogen #PA5-106312, 1:500). Uncropped images of membranes are provided in the Supplementary Figure S10.

## RESULTS

### Germline genes have a specific signature of chromatin marks in mouse ESCs

We previously identified a subset of 137 germline genes repressed by DNA methylation in mouse embryos (5). These genes, thereafter termed ‘gg-dko’ genes, have CG-rich promoters (intermediate or high CpG promoters, ICP or HCP respectively), gain promoter DNA methylation at implantation, and are upregulated in E8.5 *Dnmt3a/3b* double knockout (dko) embryos. Analysis of public RNA-seq data confirmed that ‘gg-dko’ genes are repressed in epiblast and somatic cells (dermal fibroblasts) and induced successively in germ cells (Supplementary Figure S1a). Interestingly, compared to post-implantation somatic cells, a number of ‘gg-dko’ genes show detectable expression in ESCs, morula and ICM (Supplementary Figure S1a), indicating that not all ‘gg-dko’ genes are fully repressed before DNA methylation establishment.

**Figure 1.**
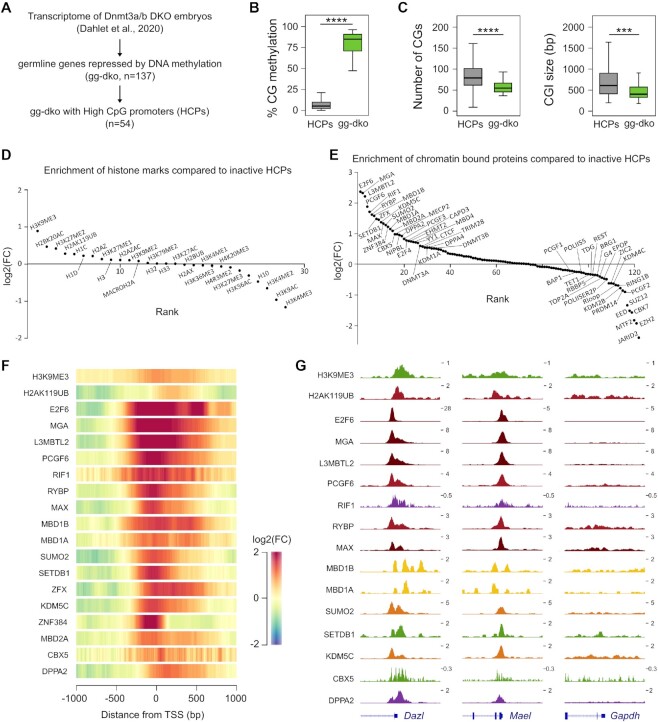
Chromatin signature of epigenetically repressed germline genes in mouse ES cells. (**A**) Diagram showing the selection of germline genes sensitive to DNA methylation in *Dnmt3a/3b* double knockout embryos (termed ‘gg-dko’). gg-dko with a HCPs were considered for the subsequent bioinformatic analysis. (**B**) Boxplots of mean CG methylation in E8.5 embryos in the promoters (–1000 to +500 bp from the TSS) of gg-dko genes with HCP compared to all HCPs. (**C**) Boxplots of the number of CGs (in –1000 to +500 bp from the TSS) and the size of predicted UCSC CpG island (CGI) associated with gg-dko genes with HCP compared to all HCPs. ****P-value < 0.0001; ***P-value < 0.001 (Mann–Whitney test). (**D, E**). Histone marks and chromatin bound proteins ranked by enrichment of ChIP-seq signal in gg-dko genes with HCP compared to all inactive HCPs in mESCs. The enrichment is represented as log2 fold change (FC). (**F**). Heatmaps showing the enrichment of ChIP-seq signal in mESCs in gg-dko genes with HCP compared to all inactive HCPs for enriched histone marks and chromatin bound proteins. The signal is represented as log2 fold change (FC) in –1000 to +1000 bp from the TSS. (**G**) Genome browser tracks of ChIP-seq signals in mESCs for histone marks and chromatin bound proteins on the promoters of germline genes *Dazl* and *Mael* and one HCP control gene *Gapdh*. UCSC RefSeq gene annotations are shown below the tracks.

To begin understanding the mechanisms of epigenetic targeting of ‘gg-dko’ promoters, we investigated if they share specific sequence or chromatin features. To reduce biases caused by varying CG richness, we focused on ‘gg-dko’ genes with HCPs (*n* = 53) and compared them to all annotated HCPs (*n* = 13693) (Figure 1A). As expected, ‘gg-dko’ HCPs have strong DNA methylation in embryos compared to all HCPs (Figure 1B). Interestingly, we found that ‘gg-dko’ HCPs have significantly lower number of CGs and shorter CGIs compared to all HCPs (Figure 1C), suggesting that lower CpG density could intrinsically contribute to lower the protection against DNA methylation. To check if ‘gg-dko’ gene promoters have a specific signature of chromatin marks, we systematically analyzed public ChIP-seq data for 28 chromatin marks in mouse ESCs cultivated in serum, including histone methylation, acetylation, ubiquitination and histone variants (Supplementary Table S1). For each dataset, we calculated the enrichment in ‘gg-dko’ HCPs compared to all HCPs (*n* = 13693). This revealed that ‘gg-dko’ HCPs are enriched for the repressive marks H3K9me3, H3K27me3 and H2AK119ub, while the most depleted marks were the active marks H3K4me3, H3K9ac, H3K79me2 (Supplementary Figure S1b). To more specifically highlight the specificity of germline genes, we next compared ‘gg-dko’ HCPs to transcriptionally inactive HCPs only (*n* = 3309). This revealed that ‘gg dko’ HCPs are enriched for H3K9me3 and H2AK119ub and depleted for H3K4me3 and H3K9ac compared to other inactive HCPs, whereas H3K27me3 is no longer enriched indicating that it is rather a general signature of inactive HCPs (Figure 1D). We conclude that HCPs of germline genes are characterized by a lower CG density, as well as higher H3K9me3 and H2AK119ub and reduced H3K4me3 compared to other inactive HCPs, which may create an environment favorable for DNA methylation.

### Germline gene promoters are bound by a distinctive set of proteins in mouse ESCs

Next, we checked whether germline genes have a distinctive signature of chromatin-bound proteins by analyzing ChIP-seq datasets for 122 chromatin modifiers in mESCs (Supplementary Table S1). ‘gg-dko’ HCPs were compared either to all HCPs (Supplementary Figure S1c) or transcriptionally inactive HCPs (Figure 1E). Interestingly, known factors involved in DNA demethylation (PRDM14, TDG, TET1) were depleted in ‘gg-dko’ HCPs compared to all HCPs or inactive HCPs, as well as R-loops and G-quadruplex (G4) structures (Figure 1E and Supplementary Figure S1c, d), in agreement with their role in opposing DNA methylation of CpG islands (28–30). KDM2B, known to protect CpG islands against methylation (31), was also depleted, as well as the H3K9 demethylases KDM4C (Figure 1E). Strikingly, the PRC2 proteins EED, SUZ12, EZH2, JARID2, MTF2 and EPOP were strongly depleted in ‘gg-dko’ HCPs (Figure 1E and Supplementary Figure S1d), indicating that lack of PRC2 recruitment is a feature of germline genes like other non-canonical PRC1 targets.

Conversely, we found as expected a strong enrichment for the PRC1.6 members E2F6, MGA, L3MBTL2, PCGF6, RYBP, MAX, the PRC1.6 cofactor RIF1 (32), the DNA methyltransferases DNMT3A and DNMT3B, as well as SETDB1, TRIM28 and CBX5 (Figure 1E, F and Supplementary Figure S1c, d). Interestingly, methyl-CpG binding domain (MBD) proteins were enriched in ‘gg-dko’ HCPs (MBD1B, MBD1A, MBD2A, MECP2 and MBD4) except MDB3 (Figure 1E, F), in agreement with MBD3 being the only MBD showing no visible preference for DNA methylation in ESCs (33). Among the top enriched factors, we also found the H3K9 methylase EHMT2/G9A and the H3K4 demethylase KDM5C known to be involved in the developmental silencing of germline genes (34,35), and SUMO2 known to participate in epigenetic repression in mouse ESCs (36). Additional enriched factors were DPPA2, DPPA4, ZFX and ZNF384 (Figure 1E, F). The Figure 1G shows binding profiles of enriched factors at the germline genes *Dazl* and *Mael* compared to the housekeeping gene *Gapdh*. This analysis shows that germline genes are bound by a specific set of chromatin modifiers and proteins in mESCs and identifies potential pathways involved in their epigenetic repression.

### A CRISPR-cas9 screen reveals candidate repressors of germline genes in mouse ESCs

**Figure 2.**
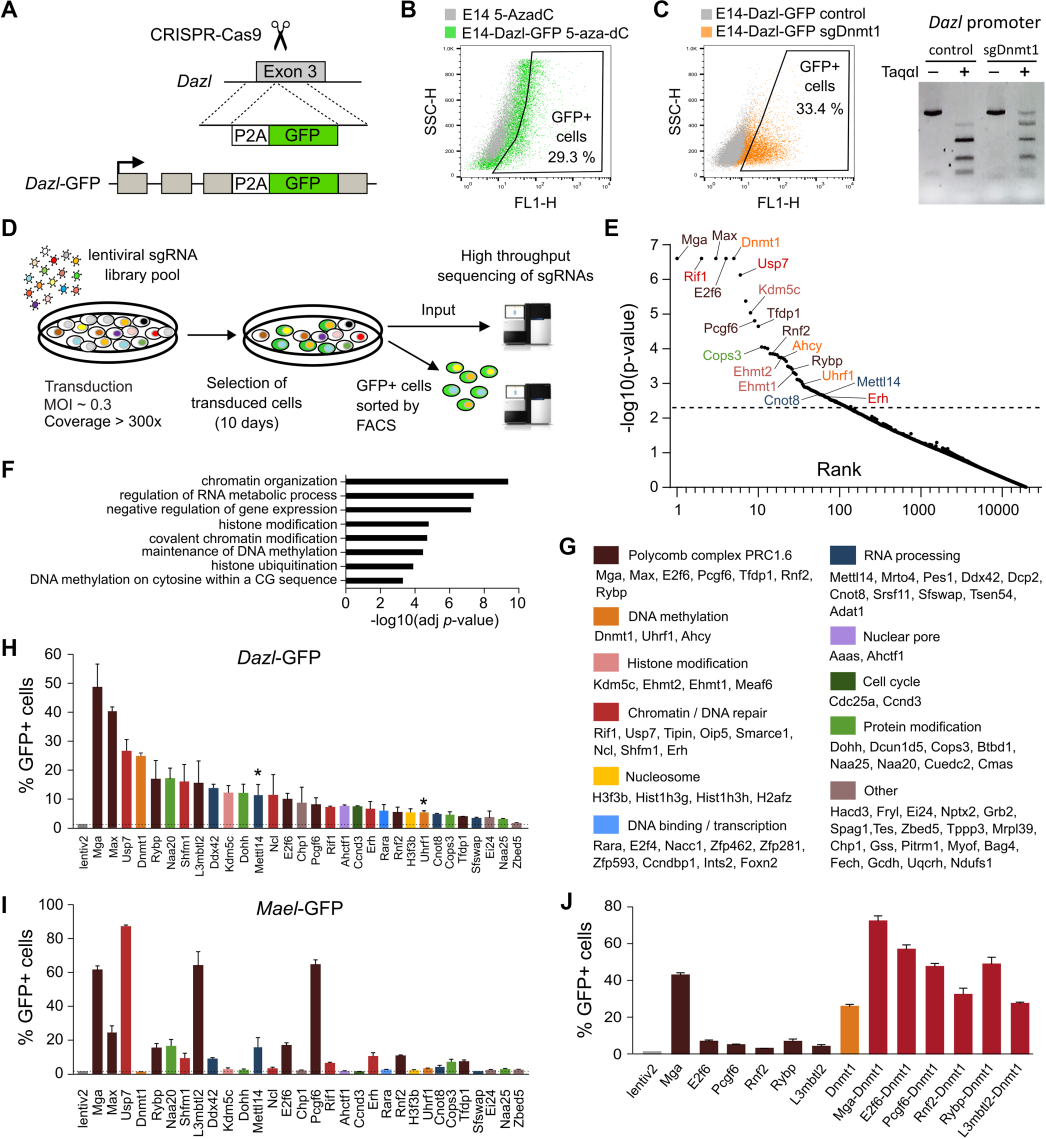
A genome-wide CRISPR screen identifies repressors of *Dazl* and *Mael* in mouse ES cells. (**A**) Knock-in strategy to insert the GFP gene into the third exon of the endogenous *Dazl* gene. (**B**) Treatment of the *Dazl-*GFP reporter line with 5-aza-dC (0.5 μM) for 2 days induces GFP fluorescence as measured by flow cytometry. Native E14 ES cells treated with 5-aza-dC were used as a control. (**C**) Infection of the *Dazl-*GFP reporter cell line with lentiviruses coding for Cas9 and a sgRNA targeting *Dnmt1* induces GFP fluorescence as measured by flow cytometry after 10 days of puromycin selection. The reporter cell line infected with Cas9 without gRNA was used as negative control. The COBRA experiment shown on the right indicates demethylation of the *Dazl* promoter in the sgRNA *Dnmt1* condition. (**D**) Principle of the genome-wide CRISPR Cas9 screen. *Dazl-*GFP cells expressing Cas9 were infected with a genome-wide lentiviral sgRNA library (Brie) and selected for 10 days before isolating GFP+ cells by flow cytometry and high-throughput sequencing of sgRNAs. (**E**) Results of the screen obtained by MAGeCK analysis. The graph shows the candidate genes ranked by their *P*-value. The names of the top candidate genes are indicated. (**F**) Top enriched GO terms of the candidate genes from the screen. (**G**) List of candidate genes from the screen grouped by molecular function. (**H**) Validation of candidate genes. *Dazl*-GFP ESCs were transduced with lentiviruses coding for Cas9 and a gRNA targeting each candidate gene. The graph shows the percentage of GFP+ cells measured by flow cytometry after 8 days of puromycin selection (mean ± SEM, *n* = 3 independent experiments). Asterisks indicate values measured at day 6 due to cell death. Lentiv2 is the control condition with a lentivirus coding for Cas9 without a gRNA. (**I**) Validation of candidate genes in *Mael*-GFP ESCs, performed as in h except that values were measured after 6 days of puromycin selection. (**J**) Percentage of GFP + cells following single or combined inactivation of PRC1.6 genes and *Dnmt1* in *Dazl*-GFP ESCs (mean ± SEM, *n* = 3 independent experiments). Values were measured by flow cytometry at day 6 of puromycin selection.

To define the factors causally involved in the silencing of germline genes, we developed a CRISPR-Cas9 knockout screening approach in mouse E14TG2a ESCs. To this end, we created a reporter cell line expressing GFP under the control of the endogenous promoter of the germline gene *Dazl* by inserting a 2A peptide and the GFP gene in the third exon of *Dazl* using CRISPR-mediated homology-directed repair (Figure 2A). We selected a clone with homozygous insertion of GFP in both *Dazl* alleles and validated the correct insertion of the p2A-NLS-GFP sequence into the *Dazl* locus by PCR and DNA sequencing (Supplementary Figure S2a–c). To validate the reporter clone, we treated it with the demethylating agent 5-aza-2-deoxycytidine and observed a strong induction of GFP fluorescence (Figure 2B). Furthermore, CRISPR-Cas9 knock-out of *Dnmt1* in the *Dazl*-GFP clone induced GFP fluorescence concomitantly with demethylation of the *Dazl* promoter (Figure 2C), which validates the sensitivity of the reporter clone for CRISPR-Cas9 screening.

We performed a genome-wide CRISPR-Cas9 knockout screen with the optimized Brie library containing 78 637 gRNAs targeting 19 674 mouse genes in lentiviral vectors (37). The *Dazl*-GFP clone was first transduced with a Cas9-Blasticidin lentivirus and selected for blasticidin resistance for 10 days. These cells were then transduced with the gRNA pooled library in lentiGuide-Puro and selected with puromycin for 10 days before isolating GFP + cells by cell sorting (Figure 2D, Supplementary Figure S2d). The screen was performed in triplicate and the enrichment of gRNAs in GFP + cells compared to input cells was analyzed by high throughput sequencing followed by statistical analysis using MAGeCK2.3 (26). Among the top 10 ranked genes were *Dnmt1* and several members of the PRC1.6 complex (*Mga*, *Max*, *E2f6*, *Pcgf6* and *Tfdp1*) (Figure 2E), which validates the screening strategy. We selected the top 100 target genes enriched in the GFP-positive cells with a *P*-value <0.005. To account for possible experimental variations caused by gRNAs that target essential genes, we also selected the top 20 genes enriched in the analysis of 2 out of 3 replicates. Finally, we filtered out genes with low expression in mESCs, which led to a list of 76 candidate genes (Supplementary Table S4). The candidate genes from the screen were enriched in biological processes related to RNA metabolism, regulation of transcription, DNA methylation and chromatin organization (Figure 2F, Supplementary Table S4).

As expected, we recovered the regulators of maintenance DNA methylation *Dnmt1*, *Uhrf1* and *Ahcy* (38), members of the PRC1.6 complex (*Mga*, *Max*, *E2f6*, *Pcgf6*, *Tfdp1*, *Rybp*, *Ring1b/Rnf2*), as well as *Kdm5c* and the H3K9 methylases *Ehmt1/Glp* and *Ehmt2/G9a* (Figure 2G). Among other genes, we recovered noteworthy candidate genes involved in chromatin regulation and DNA repair (*Ncl*, *Usp7*, *Rif1*, *Shfm1/Sem1*, *Erh*, *Tipin*), genes coding for DNA binding proteins and transcription regulators (e.g. *E2f4*, *Rara*), genes regulating RNA processing and modifications (e.g. *Mettl14*, *Ddx42, Cnot8*), and genes regulating protein modifications (e.g. *Naa20*, *Naa25*, *Dohh*, *Cops3*) (Figure 2G). Many factors that we found enriched at germline genes in Figure 1 were recovered in this list (PRC1.6 components, RIF1, KDM5C, EHMT2/G9a). Yet, genes coding for MBD proteins were not enriched in the screen, suggesting lack of repressive function (39). Unexpectedly, *Setdb1* was also not recovered in the screen. To investigate this issue, we transduced *Dazl*-GFP cells with Cas9 and sgRNAs targeting *Setdb1*. We confirmed induction of GFP fluorescence by *Setdb1* inactivation (Supplementary Figure S2e) but also observed cell lethality. This suggests that the absence of *Setdb1* in the screen is either due to lethality of *Setdb1* inactivation or technical reasons.

To characterize the network of germline gene repressors, we performed interaction analysis on the 76 genes and found tight and dense interactions between many candidates, the main core network being related to PRC1.6, DNA methylation and histone modifications (Supplementary Figure S3a). Furthermore, enrichment analysis of mammalian phenotypes associated with the candidate genes revealed an enrichment in abnormal embryogenesis (Supplementary Figure S3b, Supplementary Table S4). These results indicate that the identified candidates are members of networks of genes playing crucial roles in embryonic stages.

### Validation of candidate genes from the screen

To validate the results of the screen, we transduced the *Dazl*-GFP clone with lentiviral particles coding for Cas9 and a sgRNA targeting one of 30 genes selected among the 76 hits from the screen. We also added *L3mbtl2*, another component of PRC1.6 that was not among the top hits. The transduced cells were selected with puromycin and GFP expression was analyzed by flow cytometry. gRNAs for 30 out of the 31 candidate genes induced GFP expression at varying levels, indicating a successful identification of *Dazl* repressors (Figure 2H and Supplementary Figure S2e). Cell death was observed after inactivation of several candidates such as *Cops3, Uhrf1, Cnot8, Mettl14, Erh* and *Shfm1*, which could explain the low ranking of some of these candidates.

Next, we sought to investigate if the candidates from the screen repress other germline genes than *Dazl*. To do so, we established an independent reporter system by inserting GFP under the control of the endogenous promoter of *Mael* (Supplementary Figure S4a–c), another germline genes whose repression in mESCs relies mainly on PRC1.6 rather than DNA methylation (7). Using the same lentiviral constructs, we tested the impact of knocking out candidate genes on GFP expression in this system. The inactivation of 17 out of 31 target genes resulted in GFP activation, including PRC1.6 components but also other candidates such as *Usp7* that showed the strongest GFP activation, *Rif1*, *Cops3*, *Mettl14*, *Naa20*, *Erh*, *Ddx42* and *Shfm1* (Figure 2I and Supplementary Figure S4d), validating that these candidates are repressors of germline genes.

To investigate the cooperation between PRC1.6 and DNA methylation pathways, we performed double inactivation of PRC1.6 and *Dnmt1* and monitored GFP expression in *Dazl*-GFP cells. The dual inactivation of PRC1.6 components *Mga*, *E2f6*, *Pcgf6*, *Rybp* or *Ring1b* with *Dnmt1* resulted in increased induction of GFP expression compared to the single inactivation (Figure 2J), suggesting that PRC1.6 and DNA methylation act additively to repress germline genes. Collectively, these results validate the candidates from the screen and show that germline genes are repressed by multiple mechanisms that can act additively in mouse embryonic stem cells.

### *Usp7* is a potent repressor of germline genes in mouse ESCs

We then focused on one of the top candidates, *Usp7*, which had not yet been reported as a repressor of germline genes. *Usp7* encodes a deubiquitinating enzyme involved in the regulation of DNA replication, DNA repair and gene expression (40–44). Interestingly, the USP7 protein is a known interactor of RING1B, PRC1.1 or PRC1.2/4 components and favors the integrity and chromatin binding of these complexes (45,46). Furthermore, USP7 interacts with DNMT1 and UHRF1 and regulates their ubiquitination status and recruitment to chromatin, however potential effects of USP7 in regulating DNA methylation remain controversial (47–52).

**Figure 3.**
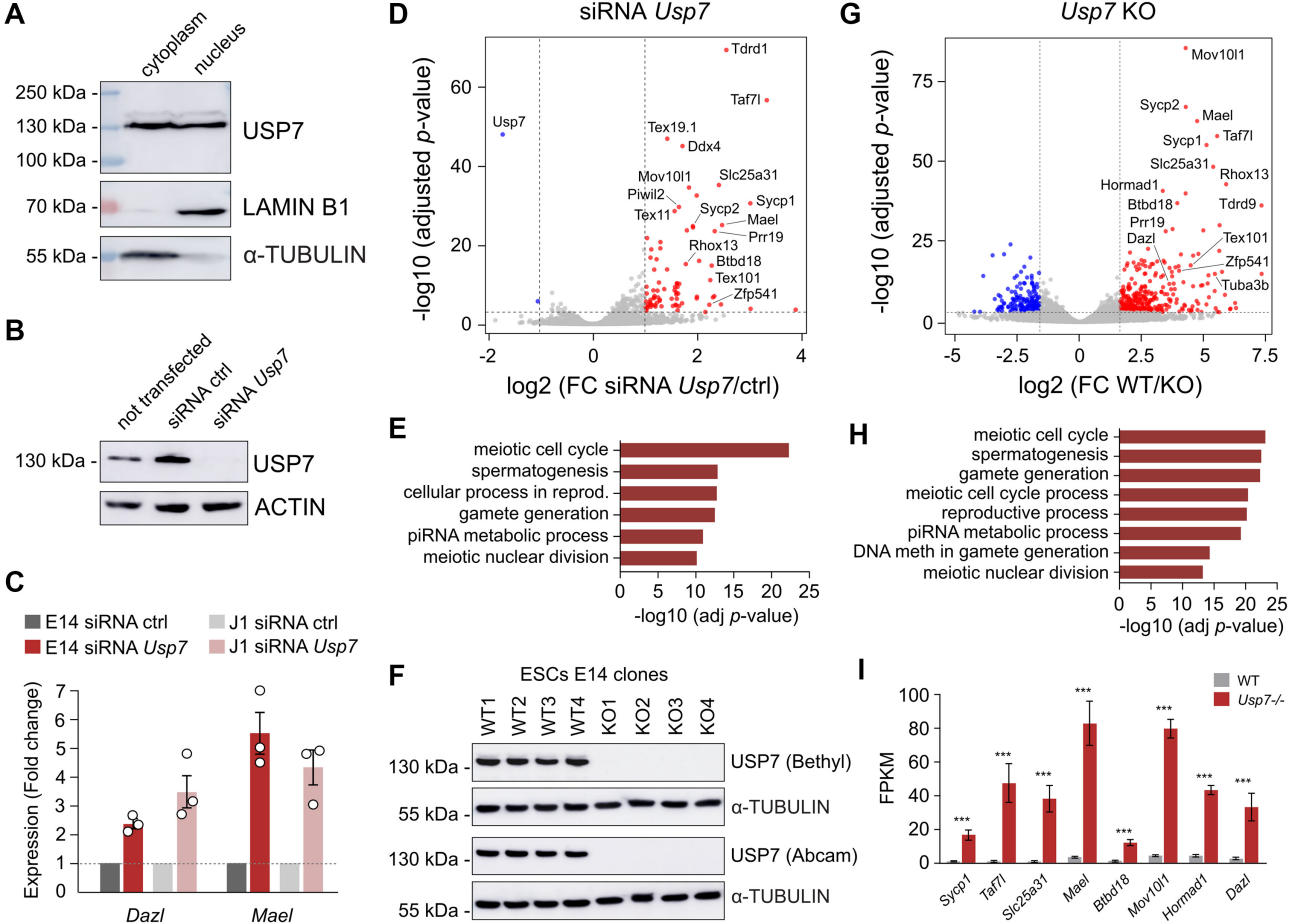
*Usp7* is a potent repressor of germline genes in mouse ES cells. (**A**) Western blot showing the abundance of USP7 in nuclear and cytoplasmic fractions of mESCs. LAMINB1 and α-TUBULIN were used as controls. (**B**) Western blot of USP7 in mESCs transfected with non-targeting control (ctrl) siRNA or *Usp7* siRNA. ACTIN was used as loading control. (**C**) Expression of germline genes *Dazl* and *Mael* measured by RT-qPCR in E14Tg2a and J1 mESCs upon siRNA knockdown of *Usp7* (72 h). The values are shown as fold change relative to non-targeting control siRNA (mean ± SEM, *n* = 3 independent experiments, expression normalized to *B2m, Gusb* and *Rpl13a*). (**D**) Volcano plot showing differentially expressed genes in *Usp7* siRNA mESCs. Significantly upregulated genes are highlighted in red. The names of the most upregulated germline genes are shown. (**E**) Top enriched gene ontology terms with their adjusted *P*-values associated with genes upregulated in *Usp7* siRNA mESCs. (**F**) Western blot showing the absence of the USP7 protein in *Usp7-/-* mESC clones using two different USP7 antibodies. α-TUBULIN was used as a loading control. (**G**) Volcano plot showing differentially expressed genes in *Usp7-/-* mESCs. Significantly upregulated and downregulated genes are highlighted in red and blue, respectively. The names of the most upregulated germline genes are shown. (**H**) Top enriched gene ontology terms with their adjusted *P*-values associated with genes upregulated in *Usp7-/-* mESCs. (**I**) Expression levels (shown as FPKM values) of germline genes strongly upregulated in *Usp7-/-* compared to WT mESCs (mean ± SEM, *n* = 4 WT and 4 *Usp7-/-* clones). ***P< 0.001 (adjusted *P*-values from DESeq2).

Using cell fractionation, we found that the USP7 protein is present both in the cytoplasm and nucleus of mESCs (Figure 3A). To study the role of *Usp7* in transcription, we performed loss of function experiments by transfecting mESCs with siRNAs targeting *Usp7* followed by RNA-seq. The efficiency of *Usp7* knockdown (kd) was validated at the transcript level (Supplementary Figure S5a) and by Western blot (Figure 3B). Following a time-limited treatment, *Usp7* knockdown did not impair cell proliferation (Supplementary Figure S5b). Furthermore, we confirmed the expected upregulation of *Dazl* and *Mael* transcripts in *Usp7*-kd cells (Figure 3C). RNA-seq identified 81 significantly up-regulated genes (fold change > 2; adjusted *P*-value < 0.001) upon *Usp7* knockdown compared to non-targeting control siRNA (Figure 3D, Supplementary Table S5). Gene ontology (GO) enrichment analysis showed that these genes are exclusively enriched for biological processes related to germline functions (Figure 3E). Indeed, among the 81 upregulated genes, 35 are known germline-specific genes with 23 belonging to the ‘gg-dko’ list (Supplementary Table S5). To exclude any cell background bias, we also conducted siRNA mediated knockdown of *Usp7* in J1 ESCs and observed a robust activation of *Dazl* and *Mael* expression in these cells at levels similar to E14TG2a ESCs (Figure 3C). Finally, to validate our results in ESCs in a more homogenous naïve state, we reanalyzed recent transcriptome data in *Usp7*-kd ESCs grown in 2i conditions (53) and observed derepression of a highly overlapping set of germline genes (Supplementary Figure S5h, i), indicating that *Usp7* also represses germline genes in mESCs under naïve conditions.

To study the consequences of long-term inactivation of *Usp7* in mESCs, we generated four independent *Usp7-/-* clones by CRISPR-Cas9. The successful inactivation of *Usp7* in KO clones was validated by Western blot using two different antibodies (Figure 3F) and sanger sequencing (Supplementary Figure S6a). *Usp7-/-* clones showed normal expression of the pluripotency markers *Pou5f1*, *Nanog* and *Sox2* but unusual morphologies of colonies (Supplementary Figure S6b, c). RNA-seq analysis in *Usp7-/-* clones compared to 4 WT clones identified 358 upregulated genes (Figure 3G, Supplementary Table S6) and GO analysis of these genes confirmed a strong enrichment for biological processes related to germline functions (Figure 3H, Supplementary Table S6). At least 68 germline genes were upregulated, many of which being among the top upregulated genes (Supplementary Table S6, Figure 3I). Furthermore, germline genes were strongly enriched among the genes commonly upregulated in *Usp7-/-* clones and *Usp7*-siRNA ESCs, indicating that they are the prime targets of *Usp7* (Supplementary Figure S6d). Altogether these experiments demonstrate that *Usp7* is a potent repressor of germline genes in mouse ESCs.

To test if *Usp7* is also required to maintain the repression of germline genes in differentiated cells, we conducted siRNA-mediated knockdown of *Usp7* in mouse embryonic fibroblasts (MEFs). In contrast to ESCs, no induction of germline genes was observed in *Usp7*-kd MEFs (Supplementary Figure S7a–c) even though germline genes can be strongly induced by the demethylating agent 5-Aza-2′-deoxycytidine in MEFs (Supplementary Figure S7c). This is reminiscent of results showing that E2F6 and MAX are required for the repression of germline genes in pluripotent cells but not in differentiated cells (13,17) and indicates that *Usp7* participates in the initiation but not long-term maintenance of silencing at germline genes.

### USP7 interacts with and promotes the stability of PRC1.6 in mouse ESCs

**Figure 4.**
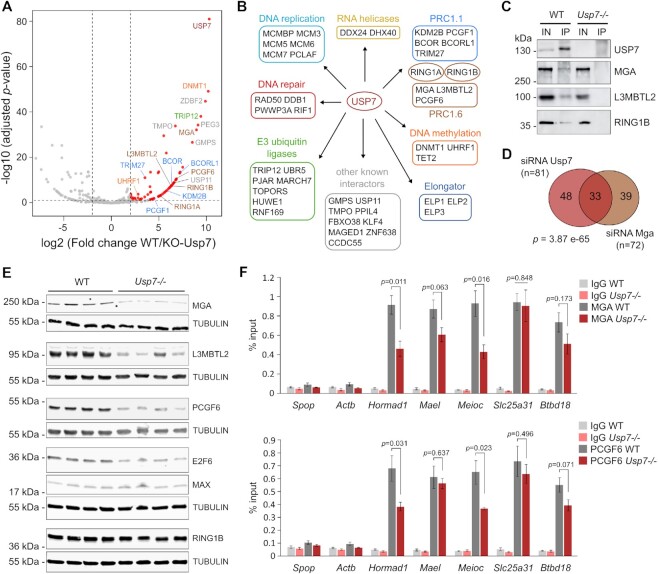
USP7 interactome analysis identifies DNMT1 and members of the PRC1.6 complex as protein partners of USP7 in mouse ES cells. (**A**) Volcano plot of USP7 interaction partners in mESCs. Anti-USP7 immunoprecipitation was performed in WT and *Usp7-/-* ESCs (*n* = 3 independent experiments). Significantly enriched protein partners (log2FC > 2, padj < 0.05) are highlighted in red. (**B**) List of selected USP7 partners grouped by molecular function. (**C**) Validation of USP7 interaction with members of the PRC1.6 complex by co-immunoprecipitation in mESCs. Anti-USP7 immunoprecipitation (IP) was performed in WT and *Usp7-/-* mESCs, followed by Western blotting on input (IN) and IP fractions with the indicated antibodies. (**D**) Venn diagram comparing the genes upregulated by siRNA knockdown of *Usp7* and *Mga* in mESCs (*P*-value: hypergeometric test). (**E**) Near-infrared fluorescence detection on the LI-COR-Odyssey imaging system of PRC1.6 proteins by western blot in WT and *Usp7-/-* mESC clones. α-TUBULIN blots were performed as loading controls. (**F**) Analysis of the binding of MGA (top) and PCGF6 (bottom) to the promoters of germline genes by ChIP-qPCR in *Usp7-/-* compared to WT mESCs (mean ± SEM, *n* = 4 clones per genotype, *P*-values: *t*-test). Primers in the *Spop* and *Actb* genes were used as negative controls.

To explore the mechanisms of action of USP7, we sought to identify its protein partners by immunoprecipitation followed by mass spectrometry in mESCs. Using an antibody directed against the endogenous USP7 protein and *Usp7-/-* ESCs as control for the mass spectrometry, we identified 82 significantly enriched USP7 interactants in WT compared to *Usp7-/-* ESCs (Figure 4A, Supplementary Table S7). Among the top hits, we identified the DNA methyltransferase DNMT1 and at a lower rank its cofactor UHRF1 (Figure 4A, B). We also confirmed many proteins previously described as USP7 interactants in other cell types: USP11 (54), GMPS (55), the E3 ubiquitin ligases TRIP12, RNF169, HUWE1, MARCH7 (56–60), members of the PRC1.1 complex KDM2B, BCOR, BCORL1, PCGF1, TRIM27 (45), the DNA replication proteins MCMBP and MCM3/5/7/6 (42), as well as PPM1G, TMPO, PPIL4, DDX24, DHX40, CCDC55, FBXO38, RAD50 (56,61–63) (Figure 4A, B).

Besides these known interactions, we establish that USP7 interacts with several members of the PRC1.6 complex in mouse ESCs: MGA, L3MBTL2, PCGF6, RING1A and RING1B (Figure 4A, B). We confirmed the interaction of endogenous USP7 with MGA, L3MBTL2 and RING1B in wild-type ESCs by immunoprecipitation followed by Western blotting, while no signal was detected in *Usp7-/-* ESCs (Figure 4C). This raises the hypothesis that *Usp7* could influence the activity of PRC1.6.

To address this hypothesis, we compared the genes repressed by *Usp7* and *Mga* using a published transcriptome of *Mga*-knockdown mouse ESCs (15). MGA is a major DNA-binding and scaffold protein of PRC1.6 and its inactivation leads to the destabilization of most PRC1.6 members (16,64), making *Mga* inhibition a good approach to probe PRC1.6 function. Knockdown of *Mga* by RNAi in ESCs resulted in the significant upregulation of 72 genes, 54 of which being known germline genes. Strikingly, 33 of these genes were in common with the genes passing the significance criteria in *Usp7*-kd ESCs (Figure 4D), and most *Mga* target genes showed a trend for upregulation in *Usp7*-kd and *Usp7*-KO ESCs (Supplementary Figure S8a). To investigate if USP7 regulates the abundance of PRC1.6, we performed Western blot of several PRC1.6 proteins in WT and *Usp7-/-* ESC clones. The results revealed unchanged levels of MAX and RING1B but reduced levels of PCGF6, MGA, L3MBTL2 and E2F6 in *Usp7-/-* compared to WT ESCs (Figure 4E and Supplementary Figure S8b). This is not associated with reduced transcript levels in *Usp7-/-* ESCs (Supplementary Figure S8b), suggesting that USP7 regulates the stability of these PRC1.6 proteins. To investigate if this leads to reduced PRC1.6 at germline genes, we carried out chromatin immunoprecipitation against PCGF6 and MGA and observed reduced binding of PCGF6 and MGA in the promoters of several germline genes in *Usp7-/-* ESCs (Figure 4F). Altogether, these results show that USP7 interacts with PRC1.6 and promotes the stability and presence at germline genes of key components of PRC1.6 in mESCs.

### *Usp7* triggers DNA methylation of germline genes

**Figure 5.**
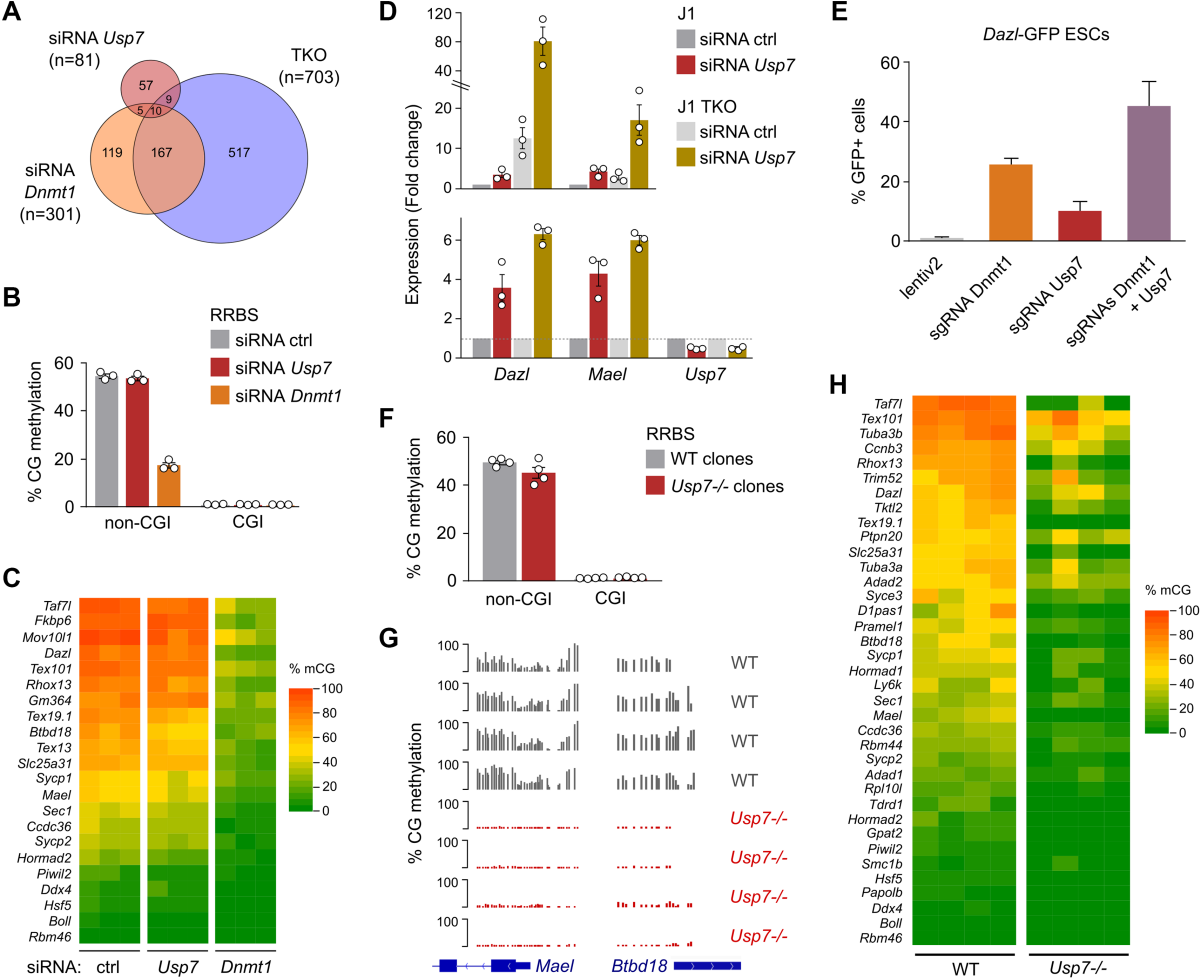
Regulation of DNA methylation of germline genes by *Usp7* in mouse ES cells. (**A**) Venn diagram comparing the genes significantly upregulated in *Usp7* siRNA, *Dnmt1* siRNA and *Dnmt-*TKO mESCs. (**B**) Quantification of CG methylation by RRBS in mESCs transfected with *Dnmt1* siRNA and *Usp7* siRNA compared to non-targeting control (ctrl) siRNA. The graph shows methylation of CGs outside of CpG islands (non-CGI) or in CpG islands (CGI) (mean ± SEM, *n* = 3 independent experiments). (**C**) Heatmap representing the percentage of CG methylation measured by RRBS in the promoters (−1000 to + 500 bp from the TSS) of germline genes in mESCs transfected with *Usp7* siRNA and *Dnmt1* siRNA compared to ctrl siRNA. (**D**) Expression of *Dazl, Mael* and *Usp7* quantified by RT-qPCR upon siRNA knockdown of *Usp7* in WT and *Dnmt-*TKO J1 ESCs (72 h). The values are represented as a fold change relative to the siRNA ctrl in WT J1 (top) or to the corresponding siRNA ctrl (bottom) (mean ± SEM, *n* = 3 independent experiments). (**E**) Percentage of GFP + cells following single or combined inactivation of *Dnmt1* and *Usp7* in *Dazl*-GFP mESCs (mean ± SEM, *n* = 3 independent experiments). (**F**) Quantification of CG methylation by RRBS in *Usp7-/-* compared to WT mESC clones (*n* = 4 WT clones, *n* = 4 *Usp7-/-* clones). (**G**) RRBS profiles in the promoters of germline genes *Mael* and *Btbd18* in WT and *Usp7-/-* clones. (**H**). Heatmap representing the percentage of CpG methylation measured by RRBS in the promoters (−1000 to +500 bp from the TSS) of germline genes in WT and *Usp7-/-* clones.

Considering that USP7 interacts with and has been proposed to regulate the activity of DNMT1 and UHRF1 (48,49) (Figure 4A, B), we investigated if *Usp7* regulates germline genes by modulating DNA methylation. First, we performed siRNA-mediated knockdown of *Dnmt1* (Supplementary Figure S5a, b) to compare the genes upregulated in *Usp7*-kd and *Dnmt1*-kd ESCs. We confirmed reduced levels of the DNMT1 protein by Western blot (Supplementary Figure S5c) and strongly reduced levels of DNA methylation genome-wide by reduced representation bisulfite sequencing (RRBS) in *Dnmt1*-kd ESCs (Supplementary Table S3, Supplementary Figure S5d-e). Of the 301 genes upregulated by *Dnmt1* knockdown (Supplementary Figure S5f, Supplementary Table S5), only 15 genes were commonly upregulated in *Usp7*-kd and *Dnmt1*-kd ESCs (Figure 5A). Furthermore, the overlap is also very poor with the genes upregulated in *Dnmt* triple knockout (TKO) ESCs (Figure 5A). This suggests that *Usp7* plays functions that are largely distinct from DNA methylation in mESCs. Indeed, *Usp7* represses several germline genes that have low promoter DNA methylation in ESCs and are not upregulated in *Dnmt1*-kd ESCs (Supplementary Figure S5g). To check whether *Usp7* regulates DNA methylation, we performed RRBS in *Usp7*-kd ESCs (Supplementary Table S3). Upon *Usp7* knockdown by RNAi, global genome methylation was not affected in contrast to *Dnmt1*-kd cells (Figure 5B, Supplementary Figure S5d, e), and no differentially methylated regions were detected. Furthermore, up-regulated germline genes did not present any notable reduction of their promoter methylation in *Usp7*-kd cells, in contrast to *Dnmt1*-kd cells (Figure 5C). These data suggest that USP7-mediated repression is decoupled from DNA methylation at germline genes at least for short-term repression.

To verify that *Usp7* regulates germline genes by DNA methylation independent mechanisms, we performed RNAi mediated inhibition of *Usp7* in *Dnmt* TKO ESCs that lack DNA methylation (25). We detected an important and additive upregulation of the germline genes *Dazl* and *Mael* after *Usp7* knockdown in TKO ESCs (Figure 5D), indicating that *Usp7* does not require DNA methylation for repression. Furthermore, the combined inactivation of *Dnmt1* and *Usp7* in the *Dazl*-GFP reporter cell line resulted in additive induction of GFP expression compared to the single inactivation (Figure 5E). These results demonstrate that *Usp7* can repress germline genes independently of DNA methylation in embryonic stem cells.

We then asked if the long-term absence of USP7 impairs DNA methylation of germline genes by performing RRBS in *Usp7* WT and KO ESC clones (Supplementary Table S3). Our results did not indicate a global loss of DNA methylation in *Usp7-/-* ESCs, although we noted some clonal variability in the methylation levels of *Usp7-/-* clones (Figure 5F). Strikingly, we observed that promoter DNA methylation of several germline genes repressed by *Usp7* was drastically reduced in *Usp7-/-* cells, as exemplified by *Mael* and *Btbd18* (Figure 5G). Quantification of DNA methylation in the promoters of up-regulated germline genes showed that most of them undergo an important decrease of promoter DNA methylation in *Usp7-/-* compared to WT ESC clones (Figure 5H and Supplementary Figure S6e). Collectively these data indicate that *Usp7* triggers in the long term the deposition of DNA methylation at germline gene promoters in ESCs. This appears as an endpoint rather than immediate cause of *Usp7*-mediated repression to sustain long term silencing.

### *Erh* and *shfm1* participate in the repression of germline genes and ERVs in mouse ESCs

We also wished to characterize the functions of two candidates from the screen: *Erh* and *Shfm1* (also known as *Sem1* or *Dss1*). These genes caught our attention because they encode short polypeptides (104 aa and 70 aa respectively) with high expression in ESCs and early embryos (Supplementary Figure S9a). Furthermore, their orthologs have been shown to mediate heterochromatin formation in yeasts. In *S. pombe*, the *Erh* ortholog Erh1 associates with Mmi1 to form the EMC complex essential for meiotic mRNA decay and assembly of facultative heterochromatin at meiotic genes (65). *Shfm1* encodes a subunit of the 26S proteasome complex implicated in DNA damage repair (66,67). In *S. cerevisiae*, the *Shfm1* ortholog maintains telomeric heterochromatin structure through modulation of histone modifications independently of the proteolytic function of the proteasome (68). These two candidates are therefore of particular interest to investigate their role in epigenetic repression in mammalian cells.

**Figure 6.**
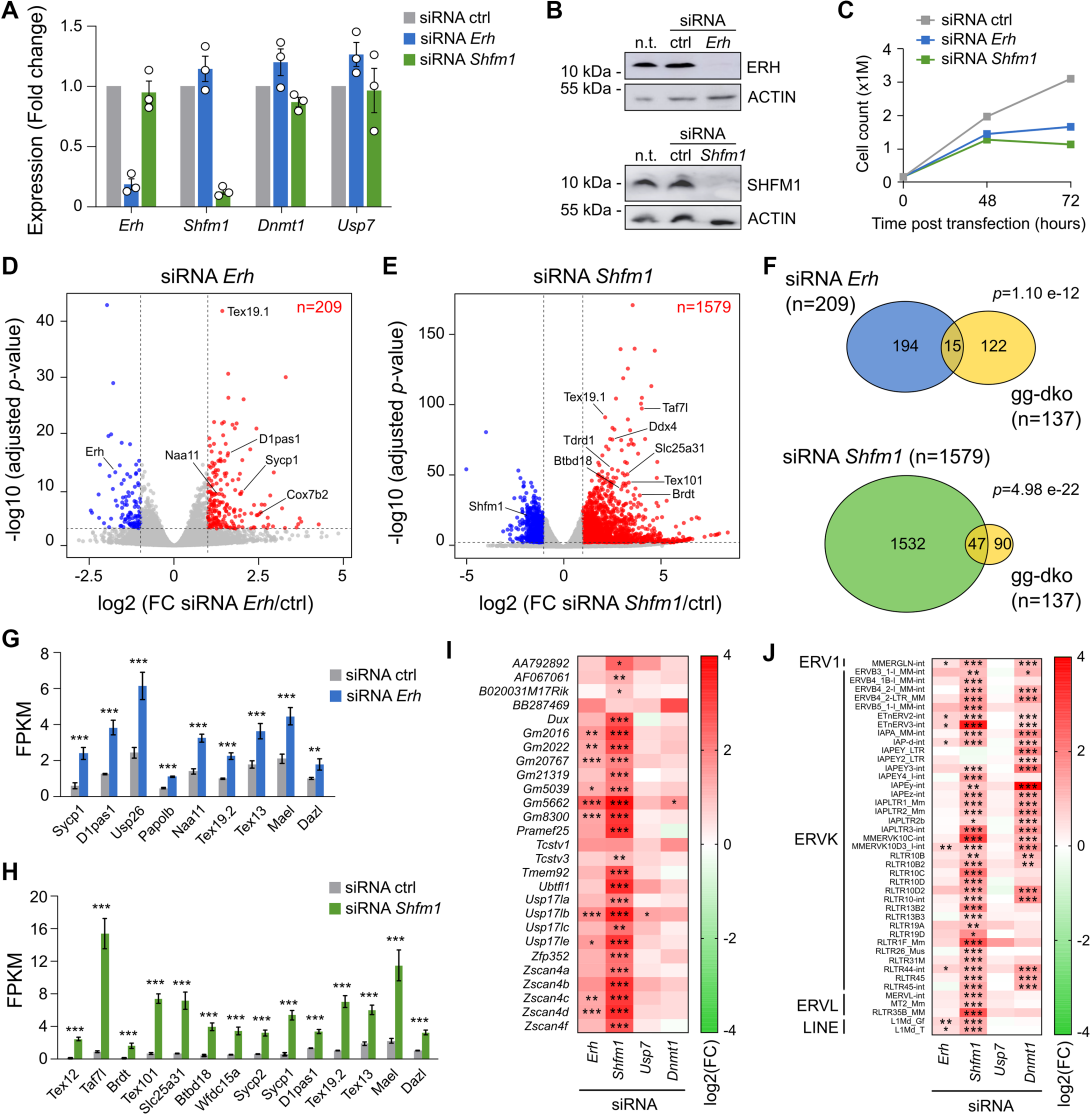
*Erh* and *Shfm1* participate in the repression of germline genes and endogenous retroviruses in mouse ES cells. (**A**) RT-qPCR analysis of *Erh*, *Shfm1*, *Dnmt1* and *Usp7* expression in mESCs transfected with *Erh* siRNA or *Shfm1* siRNA, shown as a fold change relative to non-targeting control (ctrl) siRNA (mean ± SEM, n = 3 independent experiments). (**B**) Western blot of ERH and SHFM1 in ESCs transfected with *Erh* siRNA or *Shfm1* siRNA compared to non-targeting control (ctrl) siRNA and cells not transfected (n.t.). ACTIN was used as loading control. (**C**) Cell growth curves of ESCs transfected with *Erh* siRNA or *Shfm1* siRNA compared to non-targeting control (ctrl) siRNA (mean of *n* = 3 independent experiments). (**D, E**) Volcano plot showing differentially expressed genes in *Erh* siRNA and *Shfm1* siRNA mESCs. Significantly upregulated and downregulated genes are highlighted in red and blue, respectively. The names of selected upregulated germline genes are indicated. (**F**) Venn diagrams showing the intersection of genes upregulated in *Erh* siRNA and *Shfm1* siRNA mESCs with gg-dko genes epigenetically repressed by DNA methylation (*P*-values: hypergeometric test). (**G, H**) Expression levels (shown as FPKM values) of selected germline genes upregulated in *Erh* siRNA and *Shfm1* siRNA ESCs compared to non-targeting control (ctrl) siRNA (mean ± SEM, *n* = 3 independent experiments). (**I**). Heatmap showing changes in expression of 2C-specific genes upon siRNA mediated knockdown of *Erh*, *Shfm1*, *Usp7* and *Dnmt1* in mESCs. The values represent the fold change of expression (log2) relative to the non-targeting control siRNA. (**J**) Heatmap showing changes in expression of families of transposable elements upon siRNA knockdown of *Erh*, *Shfm1*, *Usp7* and *Dnmt1* in mESCs. The values represent the fold change of expression (log_2_) relative to the non-targeting control siRNA. *P< 0.05; **P< 0.01; ***P< 0.001 (adjusted *P*-values from DESeq2 analysis).

In the screen validation experiments, we noticed that the sgRNAs against *Erh* and *Shfm1* induced lower cell counts. Furthermore, we were unable to generate KO clones for these genes by CRISPR-Cas9, suggesting that they may be essential for ESC viability. To circumvent the deleterious effect of *Erh* and *Shfm1* inactivation, we performed knockdown by RNAi in ESCs (Figure 6A). The efficient knockdown of ERH and SHFM1 was confirmed by Western blot (Figure 6B). Confirming the CRISPR-Cas9 screen validation, both knockdowns had a rapid and strong negative effect on cell proliferation as measured by cell counting (Figure 6C). Furthermore, we confirmed the upregulation of *Dazl* and *Mael* expression upon knockdown of *Erh* or *Shfm1* by RT-qPCR (Supplementary Figure S9b).

RNA-seq at 72 h post-transfection revealed 209 significantly upregulated genes (fold change > 2; adjusted *P*-value < 0.001) upon *Erh* knockdown and 1579 upon *Shfm1* knockdown (Figure 6D, E, Supplementary Table S5). Strikingly, a very high proportion of genes upregulated in *Erh*-kd cells also passed the significance criteria in *Shfm1*-kd ESCs (121/209, *P* = 1.09 e-87, hypergeometric test) (Supplementary Figure S9c), suggesting that *Erh* and *Shfm1* participate in overlapping pathways. GO enrichment analysis of upregulated genes revealed no significant term in *Erh*-kd ESCs (Supplementary Table S5), but numerous terms related to development, regulation of cell division, apoptosis and reproduction in *Shfm1*-Kd ESCs (Supplementary Figure S9d, Supplementary Table S5). When focusing on germline genes, we found that the genes upregulated in *Erh*-kd and *Shfm1*-kd ESCs significantly overlap with the ‘gg-dko’ list (Figure 6F). Furthermore, at least 18 and 61 known germline genes were upregulated in *Erh*-kd and *Shfm1*-kd ESCs respectively (Supplementary Table S5, see examples in Figure 6G, H). These results indicate that *Erh* and *Shfm1* participate in the repression of many germline genes, but the underlying mechanisms are yet to be identified. A role for *ERH* in silencing meiotic genes was also recently observed in human fibroblasts (69), indicating a conserved role of *ERH* in silencing germline genes from *S. Pombe* to higher eukaryotes.

As epigenetic regulators in embryonic cells, we wondered whether the studied candidates also repress genes specific of two-cell stage embryos (2C-genes) or transposable elements (TEs). We found that many 2C-genes such as *Zscan4* genes, *Usp17-like* genes and *Dux* are significantly upregulated by *Shfm1* knockdown, while minor effects were seen after *Erh* knockdown and no effects were observed after *Dnmt1* and *Usp7* knockdown (Figure 6I). Next, we quantified TE expression by counting reads in RepeatMasker annotations. Strikingly, *Shfm1* knockdown led to a very significant upregulation of IAPs and other ERVK families, as well as ERVL (RLTR35B_MM, MERVL-int, MT2_Mm) and L1Md elements (Figure 6J, Supplementary Table S8, Supplementary Figure S9e). In a similar way and as expected, several IAP and other ERVK families were also significantly upregulated upon *Dnmt1* knockdown (Figure 6J). In contrast, only a few TE families were modestly upregulated upon *Erh* knockdown (Figure 6J) and no significant effects were observed upon *Usp7* knockdown. We nevertheless observed a slight upregulation of MERVL-int elements in *Usp7*-kd ESCs as previously shown (40) (Figure 6J). In summary, our results describe for the first time *Shfm1* as a broad repressor involved in the silencing of germline genes, 2C-genes and ERVs in mouse ESCs.

## DISCUSSION

During development, germline genes are repressed by members of the polycomb group complex PRC1.6 and histone modifications, and are subsequently targeted by DNA methylation during the de *novo* methylation wave, which leads to their long-term repression in somatic lineages (13,20). Due to this particularity and because most CG-rich promoters are protected from DNA methylation in development, germline genes represent a paradigm for investigating the mechanisms of epigenetic targeting and repression in mammalian cells. Yet our understanding of these mechanisms remains incomplete. In this study, we used ES cells cultured in medium supplemented with serum and LIF as an experimental model to explore novel mechanisms involved in the repression of germline genes.

First, we performed a meta-analysis for ChIP-seq datasets of histone modifications and proteins to identify factors enriched or depleted at the CG-rich promoters of germline genes. In agreement with previous studies (20), we highlight a specific combination of histone marks on germline gene CG-rich promoters with enrichment of H3K9me3, H2AK119ub1, and depletion of H3K4me3. The retrieving of PRC1.6 members, SETDB1, G9A and DNMTs among the top enriched factors validates this approach. In contrast, we show that germline genes are depleted for factors involved in promoting DNA hypomethylation such as KDM2B, E2F1, R-loops, PRDM14, TDG and TET1. Interestingly, DPPA2 and DPPA4, recently identified as protectors of bivalent genes against DNA methylation (70,71), were not significantly depleted in germline gene promoters. To complement our meta-analysis approach in an unbiased way, we performed a genome-wide CRISPR-Cas9 knockout screening based on the activity of the endogenous promoter of the germline gene *Dazl*. This allows to identify factors causally required for germline gene repression. It should nevertheless be emphasized that this approach has limitations as it might miss candidates due to redundancy, lethality of KO cells or ineffective gRNAs. Indeed, *Setdb1* was not ranked among the top candidates, probably because of the lethality of *Setdb1-*KO ESCs (72). Likewise, we failed to identify *Atf7ip* as a top candidate, a partner of *Setdb1* recently shown to repress germline genes in mouse and human ES cells (73,74).

Based on the results of our meta-analysis and CRISPR-Cas9 screen, we propose that at least four main molecular routes cooperate to limit the expression of germline genes in ESCs at the chromatin level: repression by the polycomb complex PRC1.6, deposition of H3K9 methylation (by *Setdb1* and *G9a*), removal of H3K4 methylation (by *Kdm5c*) and facilitation of DNA methylation. The underlying sequence of germline gene promoters, notably the lower number of CGs and shorter CGIs compared to all HCPs, may also contribute to lower the protection against DNA methylation. Interestingly, we also show that the promoters of germline genes are depleted of PRC2 compared to other inactive promoters. Additionally, despite the fact that the deletion of PRC2 components is tolerated in mESCs, the screen did not retrieve members of the PRC2 complex among the top hits. This shows that PRC2 does not contribute to germline gene repression, in agreement with previous studies showing that the deletion in ESCs of *Eed*, the PRC2 subunit essential for H3K27me3 deposition, has no effect on germline genes (20). Given the documented antagonism between PRC2 and DNA methylation (75,76), it is also possible that the depletion of PRC2 contributes to DNA methylation at germline genes.

Besides chromatin factors, the screen also identified several genes involved in RNA processing, leading to the speculation that the expression of germline genes could also be controlled at the RNA level. Interestingly, one of these factors, *Mettl14*, catalyzes RNA methylation of ERV RNAs to promote their destabilization (77), suggesting that similar mechanisms could limit the abundance of RNA transcripts from germline genes especially during early mammalian development when the chromatin-based control is more relaxed.

Most importantly, our study led to the identification of novel factors with no previously known functions in germline gene regulation. We identified USP7, a deubiquitinating enzyme, as a potent repressor of germline genes in mouse ESCs. Despite the various described roles of USP7 (78), we show that germline genes are its principal targets in ESCs. USP7 has been reported to interact with the PRC1.1 complex and to promote H2AK119ub1 deposition by de-ubiquitinating and stabilizing RING1B (11,45,79). Our proteomics analysis, although confirming an interaction between USP7 and PRC1.1, shows that it also interacts with PRC1.6 subunits in mouse ESCs. Furthermore, we show that USP7 and PRC1.6 repress an overlapping set of germline genes, and that USP7 promotes the stability and recruitment of PRC1.6 to chromatin. These results suggest that USP7 participates in the repression of germline genes via regulating PRC1.6 activity, most probably by counteracting the ubiquitination of some PRC1.6 proteins. Interestingly, very recent studies showed that MGA, L3MBTL2 and PCGF6 are also destabilized upon USP7 inactivation in human cancer cell lines (80–82), indicating conserved USP7 function through mammalian evolution. We note however that substantial amounts of PRC1.6 remain at some germ line genes despite strong upregulation of these genes in *Usp7-/-* ESCs (Figure 4F). This suggests that USP7 might not only regulate the abundance of PRC1.6 but also its capacity to induce repression at chromatin, or represses germline genes by additional mechanisms yet to be identified.

USP7 has been suggested to regulate DNMT1/UHRF1 stability or recruitment to chromatin, and to impact maintenance of DNA methylation (48–52). While we showed an interaction of USP7 with DNMT1 and UHRF1, our results suggest no major role of *Usp7* in global genome methylation in ESCs, in agreement with other findings (47). Therefore, it remains to be determined in which physiological context the reported regulation of DNMT1/UHRF1 by USP7 is critical for global DNA methylation maintenance. Nevertheless, we demonstrate a role of USP7 in promoting DNA methylation of germline gene promoters. It is possible that USP7 regulates DNMT1 activity specifically at germline genes, or alternatively USP7 could indirectly favor the recruitment of DNA methylation by PRC1.6 (13,20).

We identify *Erh* and *Shfm1* as novel genes involved in the repression of germline genes in mouse ESCs. ERH participates in the repression of meiotic genes in *S. pombe* (65,83) and recently, the human *ERH* gene has also been shown to participate in the repression of meiotic genes in human fibroblasts (69). The identification of ERH in our study highlights a conservation of its role as a repressor of meiotic and germline genes from *S. pombe* to higher eukaryotes. The *Erh* gene encodes a small protein highly conserved in metazoans with enigmatic functions (84). ERH orthologs can be tracked to fission yeasts including *S. pombe* and *S. japonicus* but not *S. cerevisea* which lacks H3K9 methylation. Interestingly, human ERH has been predicted to be a partner of SETDB1 (85) and a recent study showed that it helps to maintain heterochromatic H3K9me3 in human cells (69). These data suggest that ERH may repress germline genes in ESCs partly by promoting H3K9me3. The underlying mechanisms connecting SHFM1 to the repression of germline genes, 2C genes and TEs also remain to be investigated. SHFM1 is a conserved, intrinsically disordered small protein with functions in protein degradation, DNA repair, transcription, and mRNA export (86). Although SHFM1 is a subunit of the 26S proteasome, several studies show that SHFM1 has proteasome independent functions (86). In particular, SHFM1 is a component of the Three prime repair exonuclease 2 (TREX-2) complex present at nuclear pore complexes and implicated in mRNA export and chromatin positioning in the nucleus (87). Given the broad function of the *Shfm1* gene in the repression of genes and ERVs, it is plausible that *Shfm1* also regulates global heterochromatin maintenance through histone or DNA methylation. Furthermore the involvement of *Shfm1* in the repression of 2C-genes and MERVL elements, which are markers of the totipotent 2-cell stage embryo (88), suggests important roles in controlling totipotency in early development.

Finally, an intriguing observation is the overlap between factors limiting the expression of germline genes and the TE silencing machinery. Indeed, in addition to SHFM1, many repressors of germline genes described in the literature or identified in this study also repress transposable elements such as SETDB1 (89), G9A (90), RIF1 (91), METTL14 (77) and USP7 (40). This may underlie an evolutionary convergence between mechanisms repressing harmful TEs and germline genes in mammalian somatic cells.

## DATA AVAILABILITY

The sequencing data generated in this study (RRBS and RNA-seq) have been deposited in the NCBI Gene Expression Omnibus (GEO) under the accession number GSE192556. The mass spectrometry proteomics data have been deposited to the ProteomeXchange Consortium via the PRIDE partner repository with the dataset identifier PXD030666. The following deposited datasets were also used: RNA-seq in E14 ESCs in serum and 2i (GSE77420), RNA-seq in morula, ICM and E6.5 embryos (GSE98150), RNA-seq and WGBS in E8.5 mouse embryos (GSE130735), RNA-seq in mouse dermal fibroblasts (GSE175615), RNA-seq in mouse PGCs (GSE76958), sperm (GSE49624) and oocytes (GSE56697), RNA-seq of *Mga* siRNA ESCs (GSE84480), RNA-seq of *Usp7* siRNA ESCs in 2i medium (GSE149341), RNA-seq of *Dnmt* TKO ESCs (GSE67867). The list of ChIP-seq datasets used in the Figure 1 is provided in the Supplementary Table S1.

## Supplementary Material

gkad071_Supplemental_Files

## Notes

Present address: Hala Al Adhami, IEO, European Institute of Oncology IRCCS, Department of Experimental Oncology, Via Adamello 16, 20139 Milan, Italy.

Present address: Anaïs Flore Bardet, Institute of Genetics and Molecular and Cellular Biology (IGBMC), CNRS UMR7104, INSERM U1258, University of Strasbourg, Illkirch, France.
