# Supplementary material for: Systematic identification of factors involved in the silencing of germline genes in mouse embryonic stem cells
Source: Nucleic Acids Res. 2023 Feb 11;51(7):3130–49. doi: 10.1093/nar/gkad071 (PMC10123117; doi:10.1093/nar/gkad071)

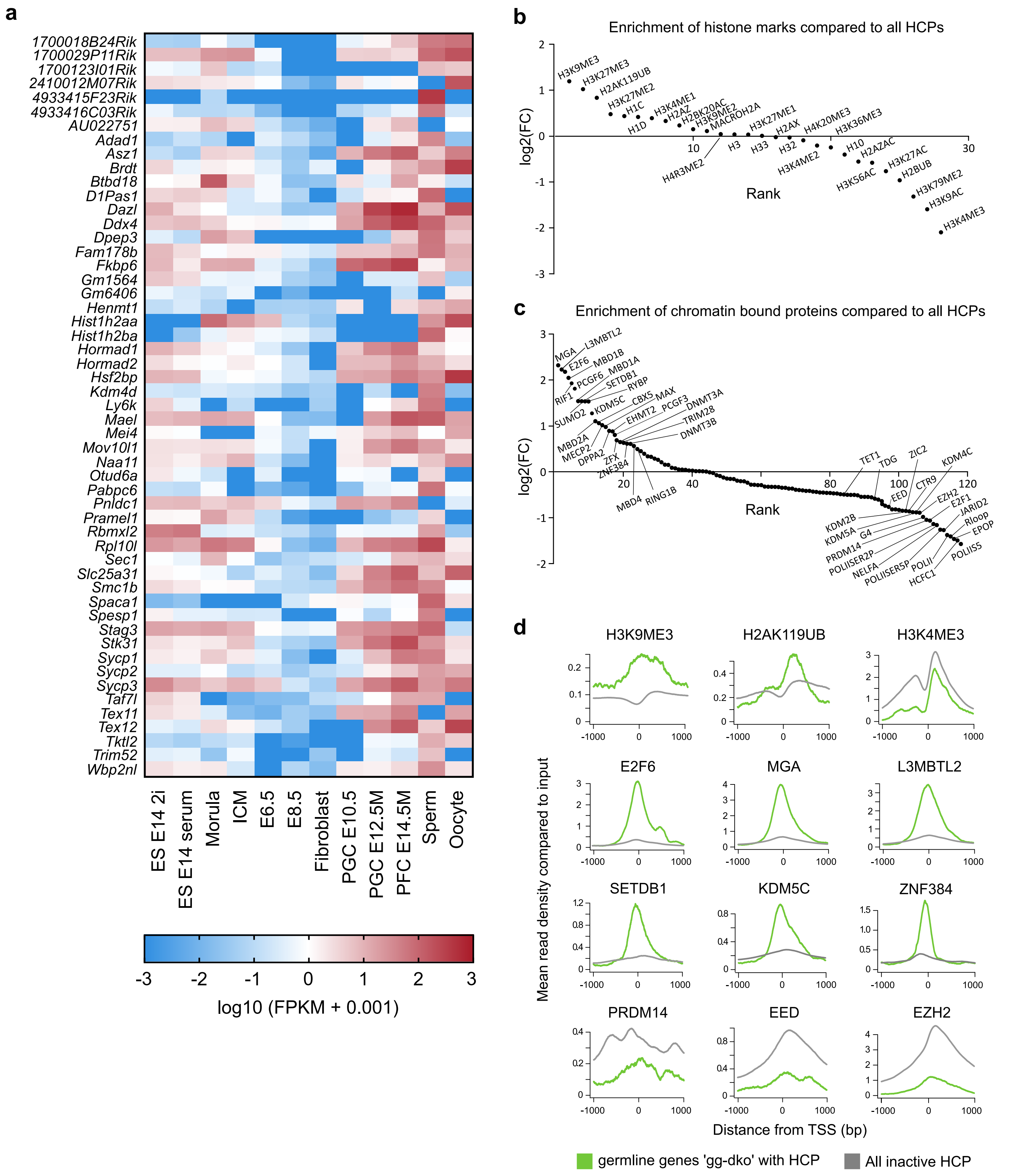

Figure S1. Expression pattern and chromatin profile of gg-dko genes. a. Heatmap showing the expression of ‘gg-dko’ germline genes with HCP (n=53 genes) in mouse ESCs, early embryos, fibroblasts and germline development using published RNA-seq datasets. **b-c.** Histone marks and chromatin bound proteins ranked by enrichment of ChIP-seq signal in gg-dko genes with HCP compared to all HCPs in mESCs. The enrichment is represented as log2 fold change (FC). **d.** Metaplots of ChIP-seq signals of selected histone marks and chromatin proteins around the promoters (-1000 to +1000 bp from the TSS) of 'gg-dko' genes with HCP (green line) compared to all inactive HCPs (grey line) in mESCs.

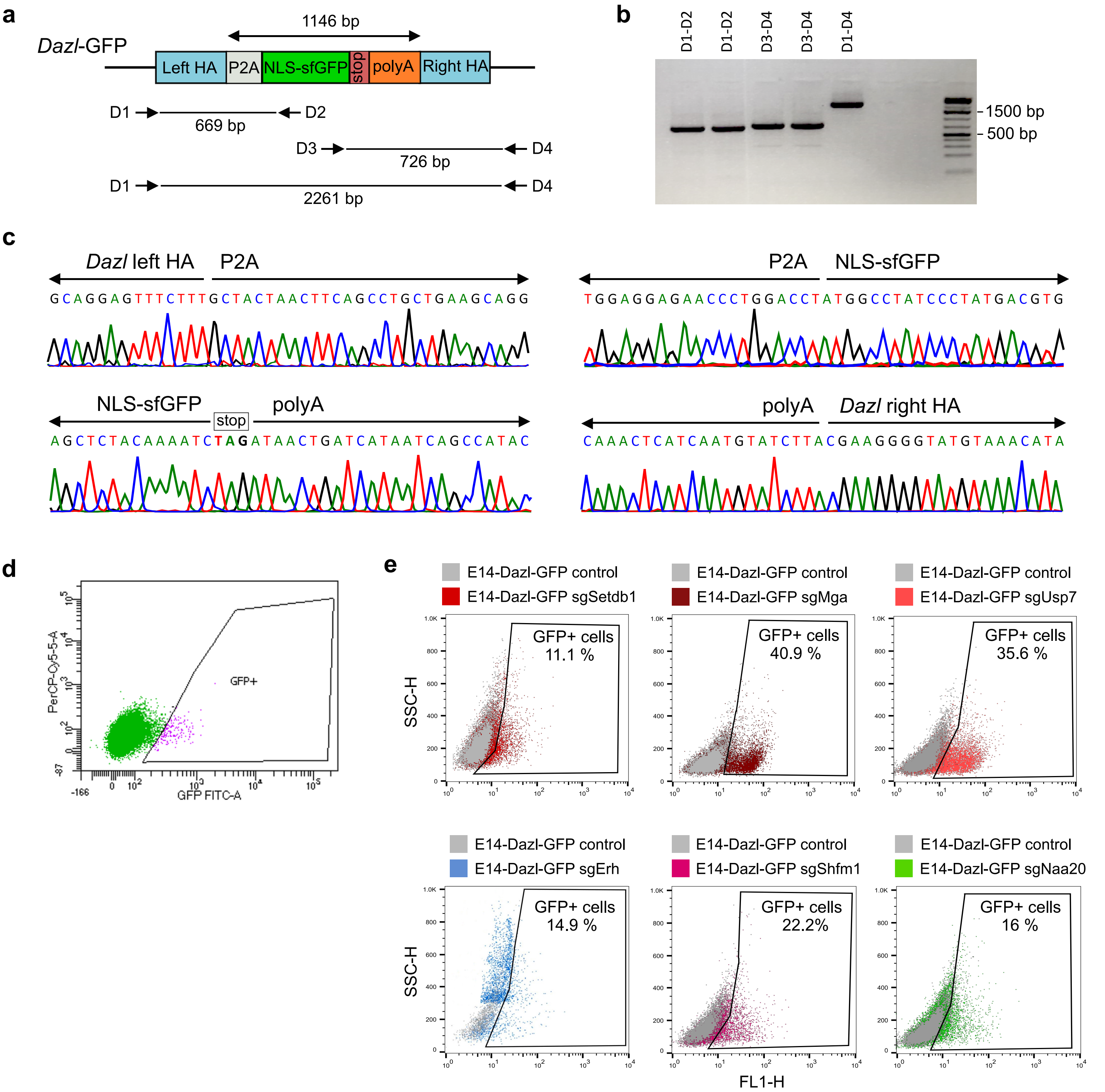

Figure S2. The *Dazl*-GFP reporter ESC line. a. Detailed scheme of the knock-in of the GFP construct flanked by homology arms (HA) by CRISPR-Cas9 in the *Dazl* exon 3 to create the *Dazl*-GFP reporter mESC line. D1 to D4 indicate the positions of primers used for genomic PCR. **b.** Genomic PCRs with primers D1 to D4 validate the insertion of the GFP sequence in the *Dazl* locus in the reporter clone. **c.** Sanger sequencing results showing the correct integration of the GFP construct in the *Dazl*-GFP reporter clone. **d.** Representative example of FACS scatter plot showing the isolation of GFP+ cells after 10 days of selection for the CRISPR/cas9 screen in the *Dazl*-GFP reporter line. **e.** Validation of selected candidate genes in the *Dazl*-GFP reporter line. Cells were infected with lentiviral particles coding for Cas9 and sgRNAs targeting indicated candidates followed by flow cytometric analysis of GFP expression after 8 days of puromycin selection. The reporter cell line infected with the same lentiviral vector without gRNA was used as negative control (shown in grey). The percentage of GFP+ cells in the sgRNA condition is indicated.

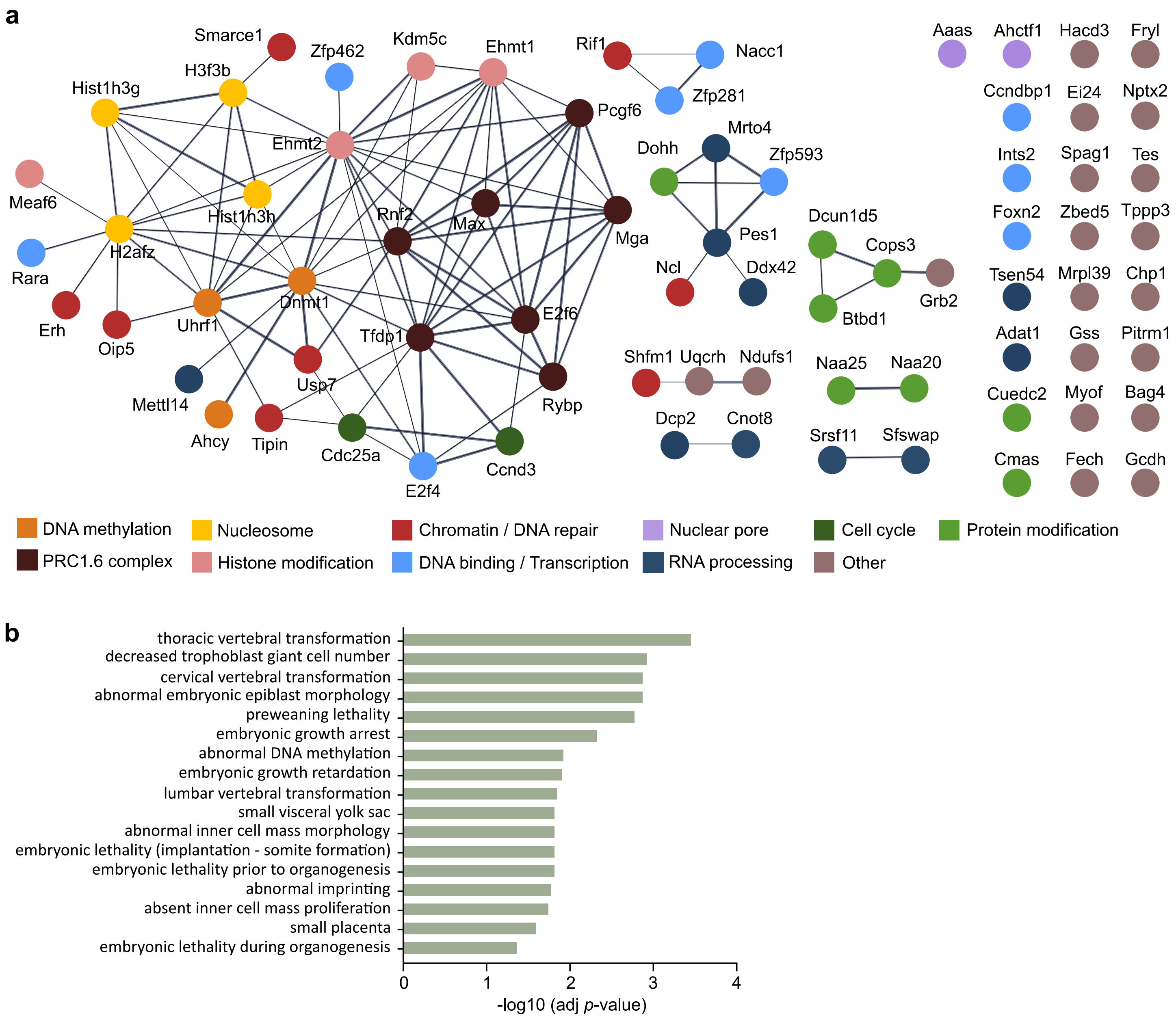

Figure S3. Network analysis of candidate genes from the screen. a. Protein-protein interaction network clustering of the 76 selected candidates using STRING. Nodes represent the candidates and the thickness of the connecting lines indicates the strength of data support related to interactions between two given nodes. **b.** Mammalian phenotypes associated with the 76 candidate genes. The top ranked phenotypes are shown with their associated adjusted p-values (-log10).

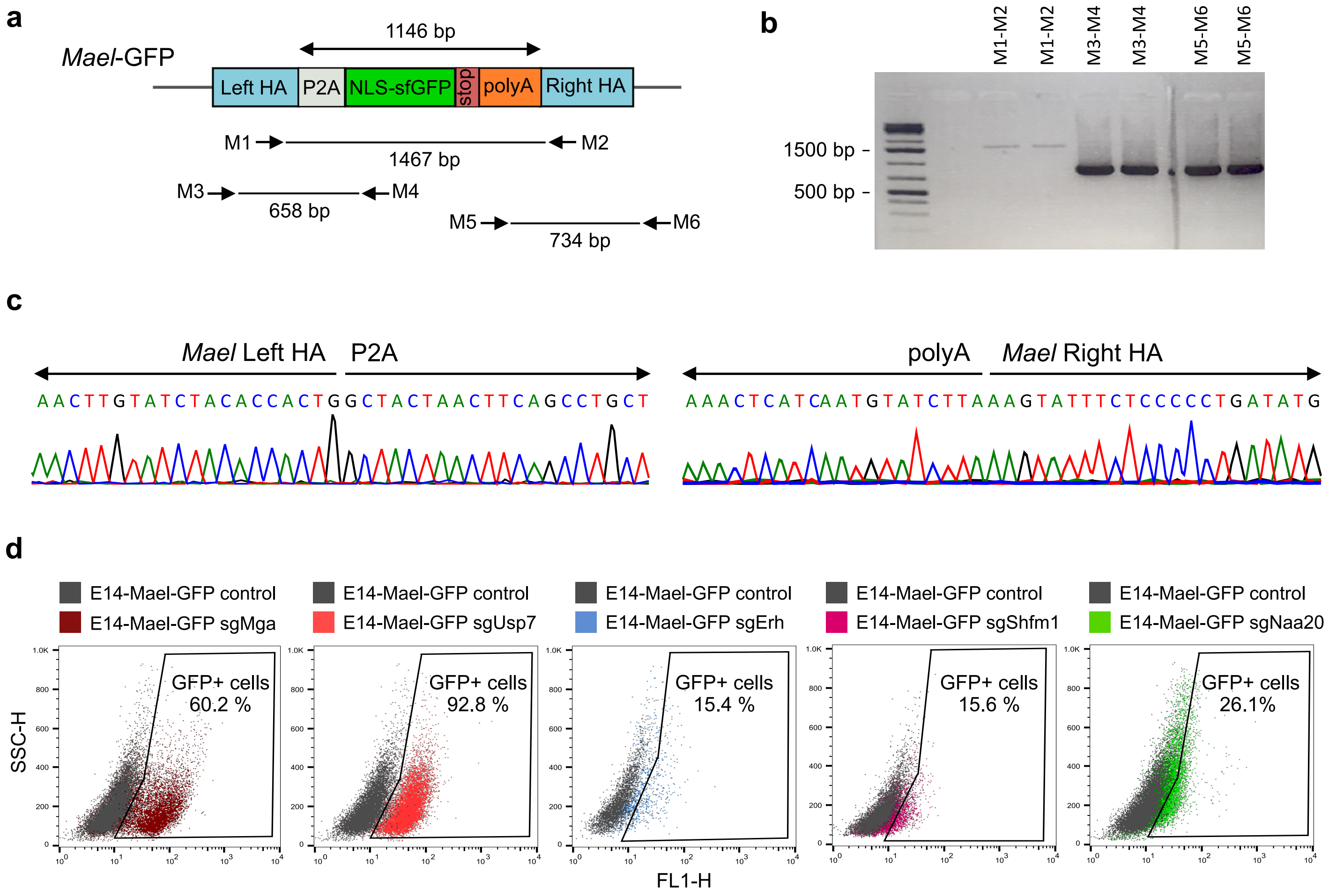

Figure S4. The *Mael*-GFP reporter ESC line. a. Detailed scheme of the knock-in of the GFP construct flanked by homology arms (HA) by CRISPR-Cas9 in the *Mael* exon 3 to create the *Mael*-GFP reporter mESC line. M1 to M6 indicate the positions of primers used for genomic PCR. **b.** Genomic PCRs with primers M1 to M6 validate the insertion of the GFP sequence in the *Mael* locus in the reporter clone. **c.** Sanger sequencing results showing the correct integration of the GFP construct in the *Mael*-GFP reporter clone. **d.** Validation of selected candidate genes in the *Mael*-GFP reporter line. Cells were infected with lentiviral particles coding for Cas9 and sgRNAs targeting indicated candidates followed by flow cytometric analysis of GFP expression after 6 days of puromycin selection. The reporter cell line infected with the same lentiviral vector without gRNA was used as negative control (shown in grey). The percentage of GFP+ cells in the different sgRNA conditions is indicated.

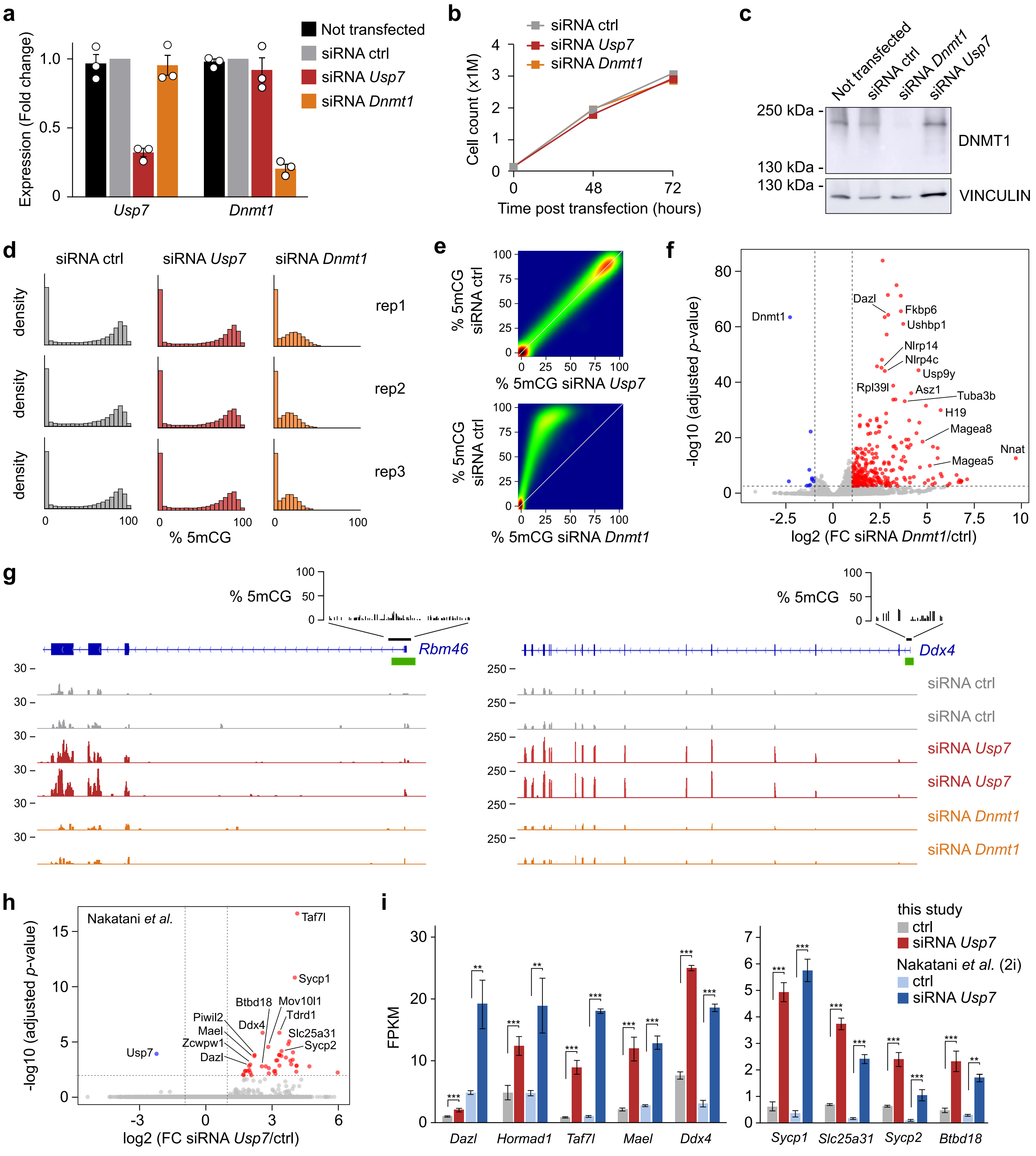

Figure S5. Validation and analysis of *Usp7* and *Dnmt1* knockdown by siRNA in mESCs. a. RT-qPCR analysis of *Usp7* and *Dnmt1* expression in mESCs transfected with *Usp7* siRNA or *Dnmt1* siRNA (72h). The graph shows fold changes relative to non-targeting control (ctrl) siRNA (mean ± SEM, n=3 independent experiments). **b.** Cell growth curves of mESCs transfected with ctrl, *Usp7* or *Dnmt1* siRNA (mean of n=3 independent experiments). **c.** Western blot of DNMT1 72h after siRNA transfection. VINCULIN was used as loading control. **d.** Genome-wide distribution of CG methylation levels measured by RRBS in 500 bp windows in mESCs transfected with ctrl, *Dnmt1* or *Usp7* siRNA. 3 independent replicates are shown per condition. **e.** Density scatter plots of CG methylation in 500 bp windows in mESCs transfected with *Dnmt1* or *Usp7* siRNA compared to ctrl siRNA (average of 3 independent experiments). **f.** Volcano plot showing differentially expressed genes in *Dnmt1* siRNA mESCs. Significantly upregulated and downregulated genes are highlighted in red and blue. **g.** Examples of germline genes with low promoter DNA methylation repressed by *Usp7* but not *Dnmt1*. RRBS results from WT mESCs are shown above the gene. Genome browser tracks of 2 out of 3 RNA-seq replicates are shown below the gene. Green rectangles depict CpG islands. **h.** Volcano plot showing differentially expressed genes following *Usp7* knockdown by siRNA in mESCs cultured with 2i inhibitors (data from Nakatani et al.). **i.** Expression of germline genes commonly derepressed by *Usp7* siRNA in standard (this study) and 2i medium (Nakatani *et al.*) (mean ± SEM, n=2 or 3 independent experiments). ** $p<0.01$; *** $p<0.001$ (adjusted *p*-values from DESeq2).

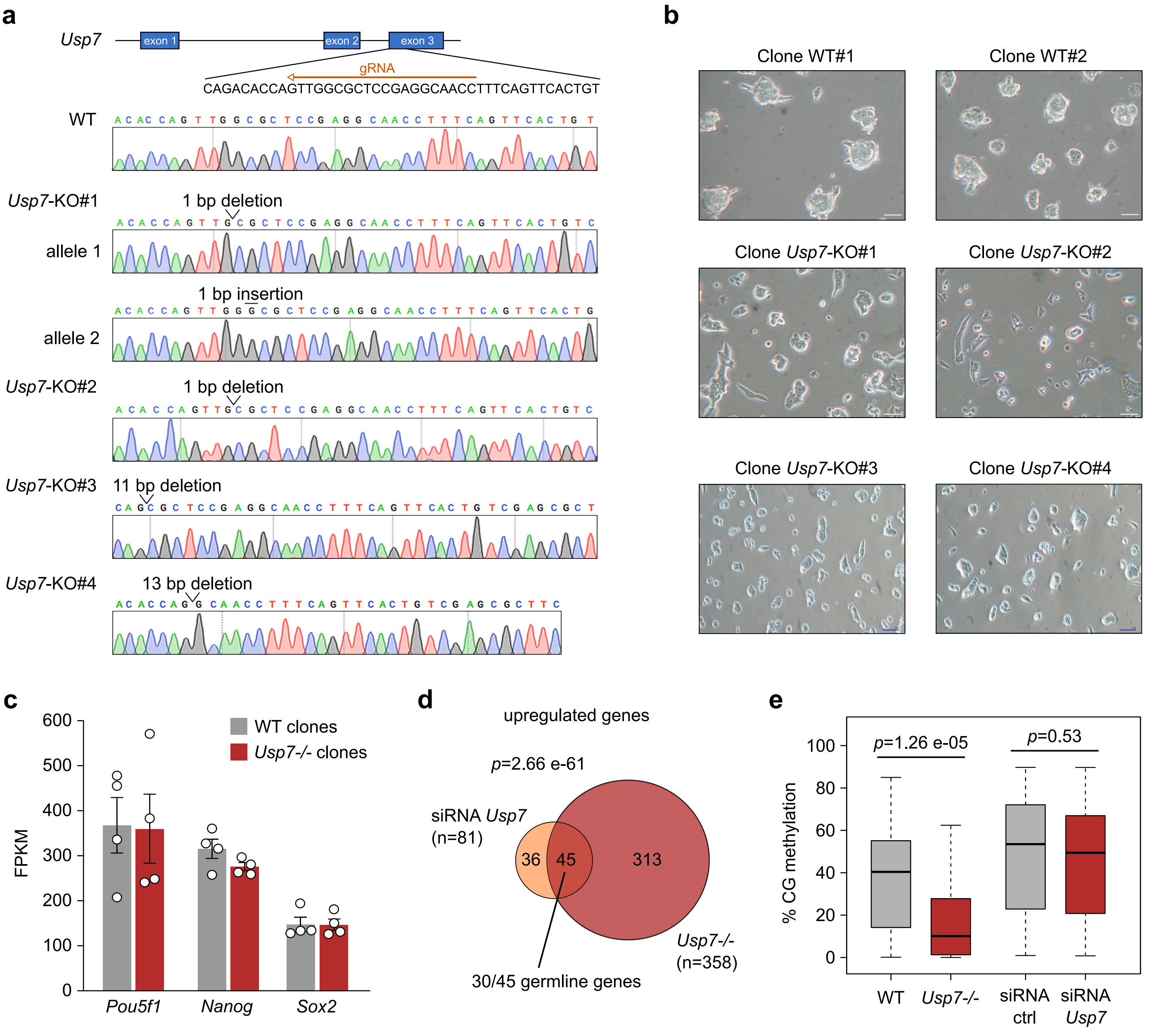

Figure S6. Characterization and analysis of *Usp7-/-* mESC clones. a. Target sequence of the sgRNA in the exon 3 of the *Usp7* gene used to create *Usp7-/-* mESC clones, and validation of the induced mutations in *Usp7*-KO clones by sanger sequencing. **b.** Phase contrast microscopy showing the morphologies of ES cell colonies in *Usp7-/-* clones compared to two WT clones. Scale bar: 50 µm. **c.** Expression (FPKM) of the core pluripotency genes *Pou5f1*, *Nanog* and *Sox2* measured by RNA-seq in *Usp7-/-* compared to WT clones (mean ± SEM, n=4 WT and *Usp7-/-* clones). **d.** Venn diagram representing the overlap between the genes significantly upregulated in *Usp7* siRNA mESCs and *Usp7-/-* mESCs (*p*-value: hypergeometric test). **e.** Quantification of promoter DNA methylation (-1000 to +500 bp from the TSS) in WT and *Usp7-/-* mESC clones (mean of n=4 clones per genotype) for germline genes significantly upregulated in *Usp7-/-* mESCs. For comparison, RRBS promoter DNA methylation of the same genes in siRNA ctrl and siRNA *Usp7* mESCs (mean of n=3 independent replicates per condition) is also shown. Promoters with at least 4 CpGs covered by RRBS are included in the analysis (n=47 genes). *p*-values: Wilcoxon test.

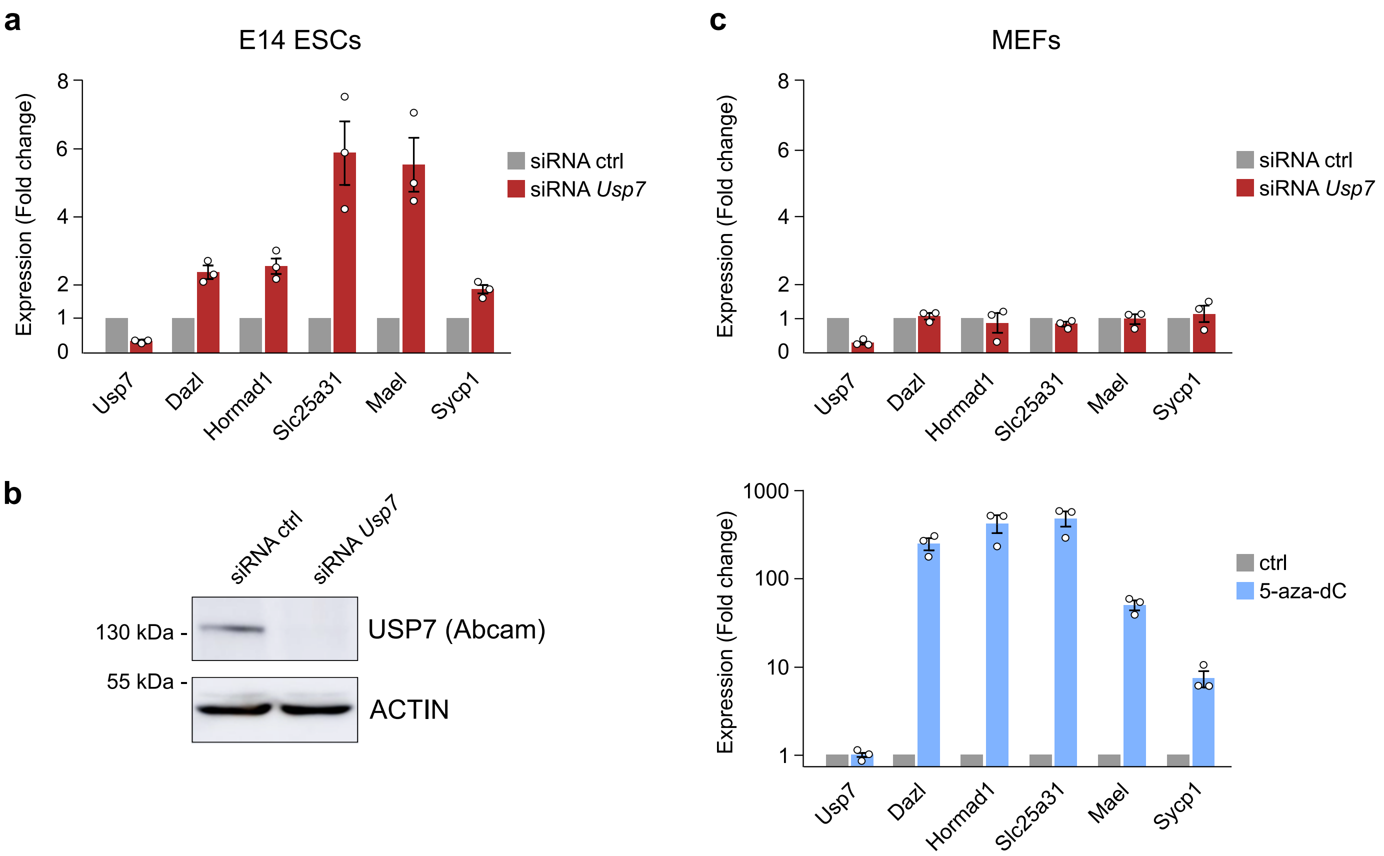

Figure S7. siRNA mediated knockdown of *Usp7* does not lead to reactivation of germline genes in MEFs. a. Expression of *Usp7* and germline genes measured by RT-qPCR after siRNA knockdown of *Usp7* (72h) in mESCs. **b.** Western blot of USP7 validates the efficiency of the knockdown in MEFs transfected with *Usp7* siRNA (72h). ACTIN was used as loading control. **c.** Expression of *Usp7* and germline genes measured by RT-qPCR after siRNA knockdown of *Usp7* (72h) in MEFs (top). As a control, expression of the same genes was measured in MEFs treated with 0.5 μM 5-Aza-2'-deoxycytidine (5-aza-dC) for 72h (bottom), which demonstrates that MEFs possess the transcriptional machinery necessary for the expression of germline genes. In **a** and **c**, the graphs show fold changes relative to the control condition (mean ± SEM, n=3 independent experiments).

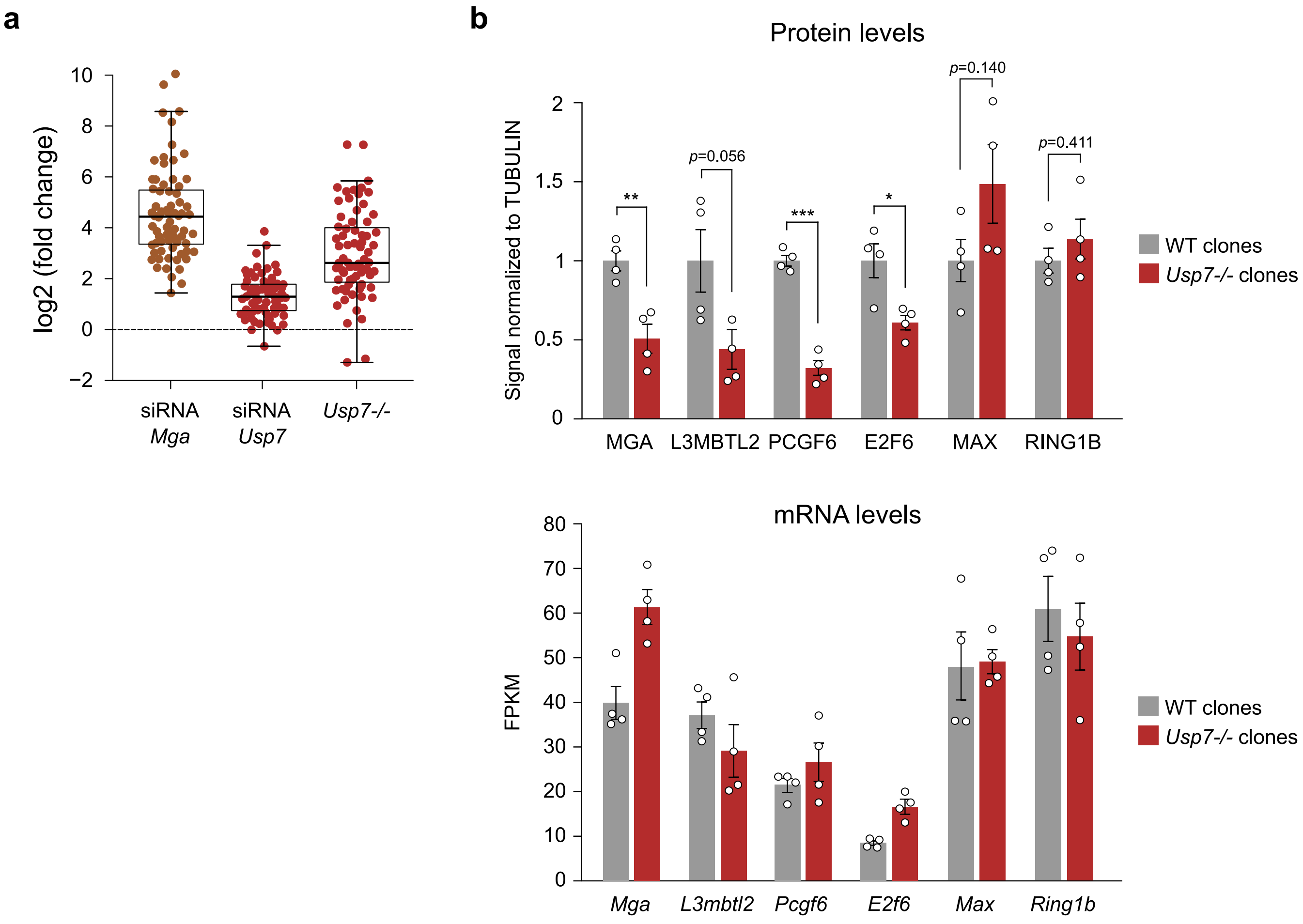

Figure S8. Interplay between USP7 and PRC1.6 in mESCs. a. Boxplot representing the fold change of expression of *Mga*-repressed genes (n=72) in *Mga* siRNA mESCs (data from Endoh *et al.*), *Usp7* siRNA mESCs and *Usp7-/-* mESCs. **b.** Top: quantification of PRC1.6 protein levels in WT and *Usp7-/-* mESC clones from the western blots shown in Figure 4e. The signal was normalized to TUBULIN and is represented as a fold change relative to the mean signal in WT clones (mean ± SEM, n=4 independent clones per genotype). *p*-values: t-test (*: $p<0.05$, **: $p<0.01$, ***: $p<0.001$). Bottom: quantification of mRNA levels by RNA-seq for the corresponding genes in WT and *Usp7-/-* mESC clones (mean ± SEM, n=4 independent clones per genotype)

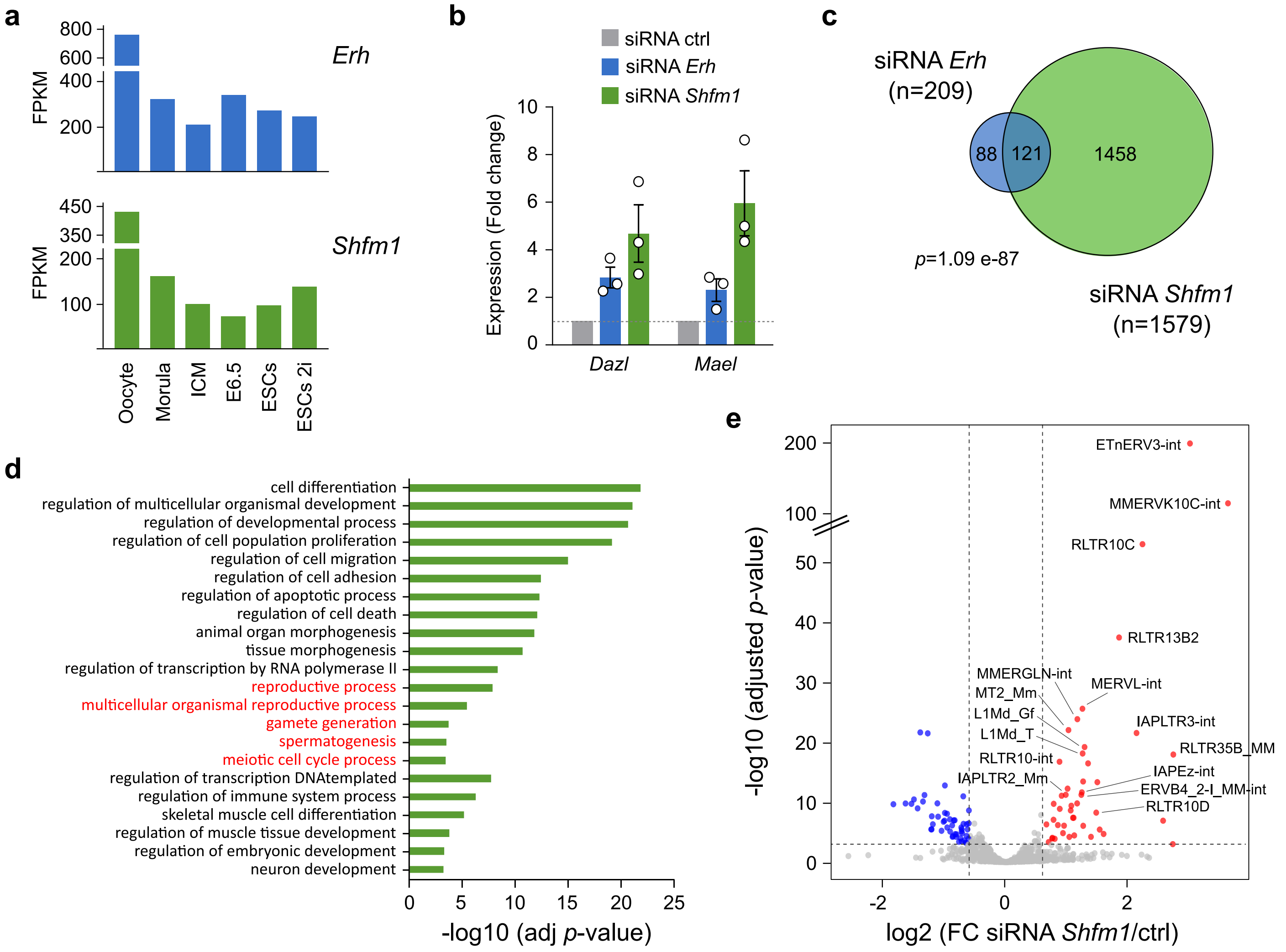

Figure S9. Consequences of *Erh* and *Shfm1* knockdown by siRNA in mESCs. a. Expression of *Erh* and *Shfm1* genes extracted from public RNA-seq datasets (plotted as FPKM) in early embryonic stages and ES cells. **b.** Expression of *Dazl* and *Mael* measured by RT-qPCR in *Erh* siRNA and *Shfm1* siRNA mESCs compared to non-targeting control (ctrl) siRNA (mean ± SEM, n=3 independent experiments). **c.** Venn Diagram showing the overlap between the genes significantly upregulated in *Erh* siRNA and *Shfm1* siRNA mESCs (*p*-value: hypergeometric test). **d.** Selection of gene ontology terms significantly enriched in genes upregulated in *Shfm1* siRNA mESCs. The full list is available in the Supplementary Table S5. GO terms related to germline genes are highlighted in red. **e.** Volcano plot representing differentially expressed transposable element (TE) families in *Shfm1* siRNA mESCs compared to non-targeting control (ctrl) siRNA. Significantly upregulated and downregulated transposable elements are highlighted in red and blue, respectively. The names of selected upregulated transposable elements are indicated.

Figure S10

Source data_uncropped Western blots for Figure 3a

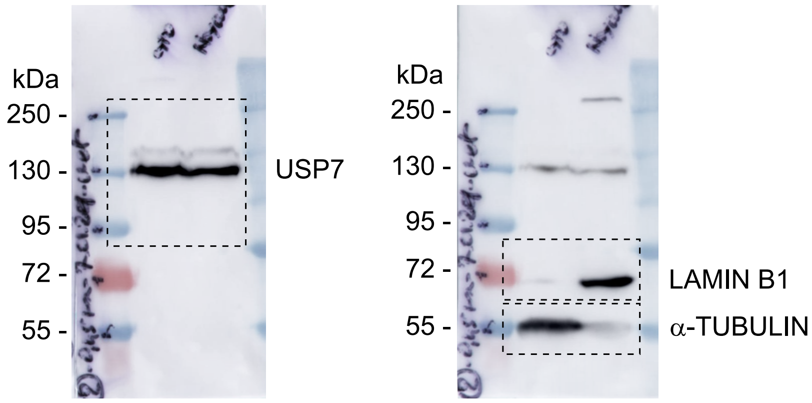

Source data_uncropped Western blots for Figure 3b

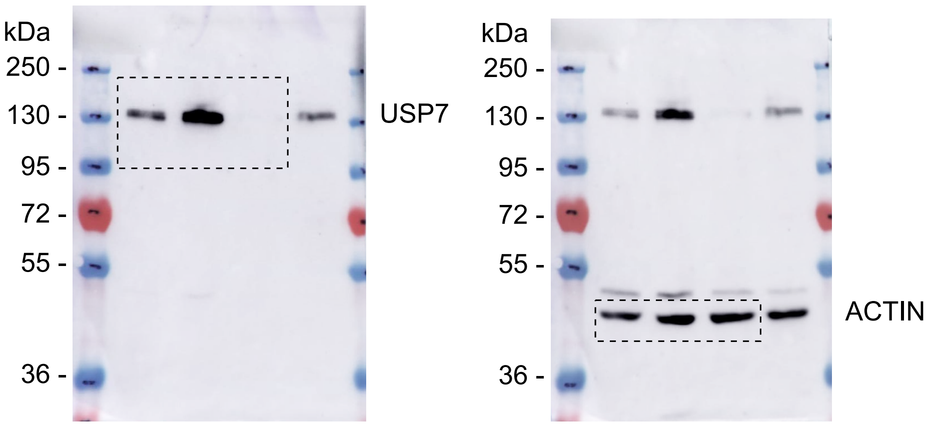

Source data_uncropped Western blots for Figure 3f

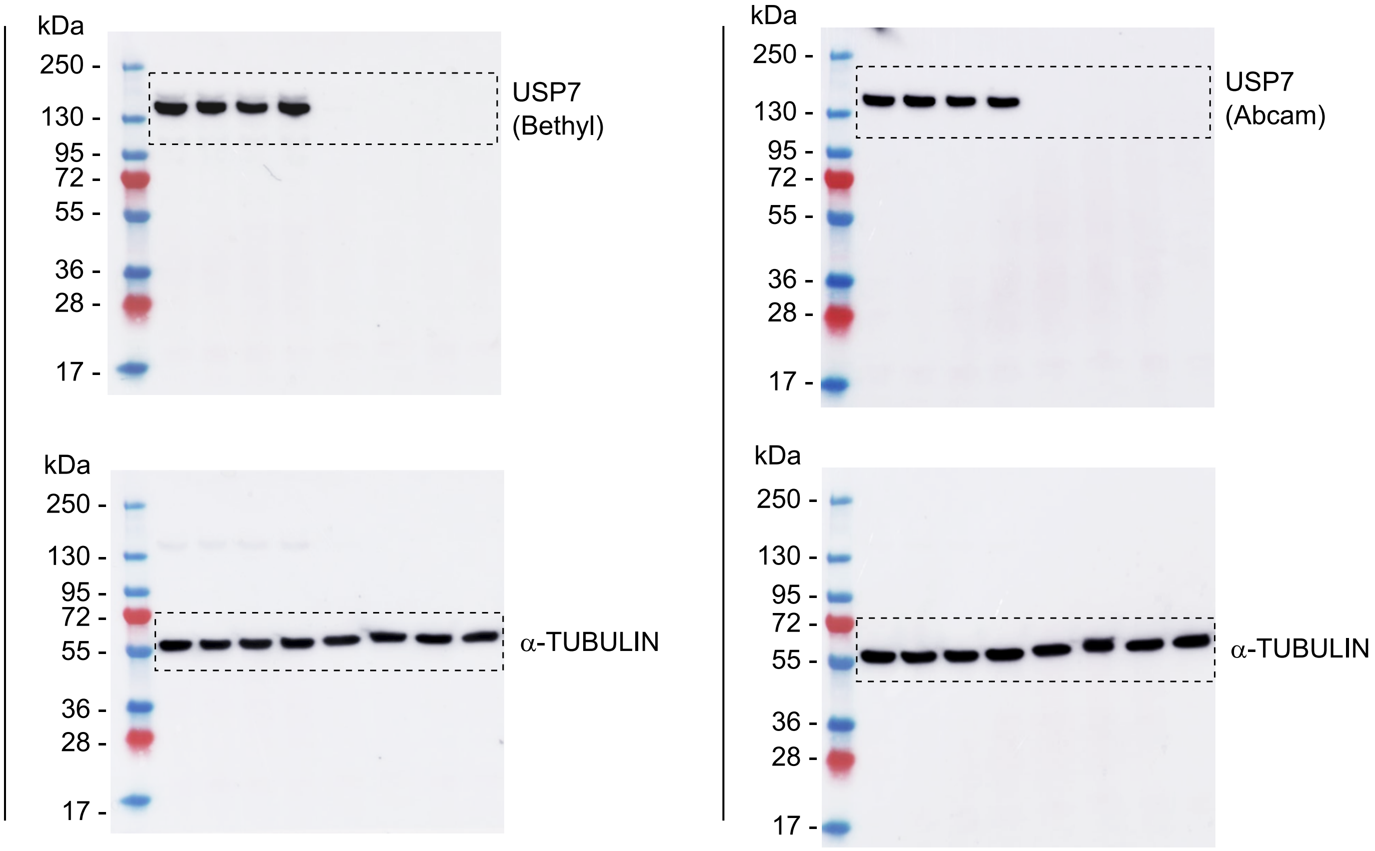

Source data_uncropped Western blots for Figure 4c

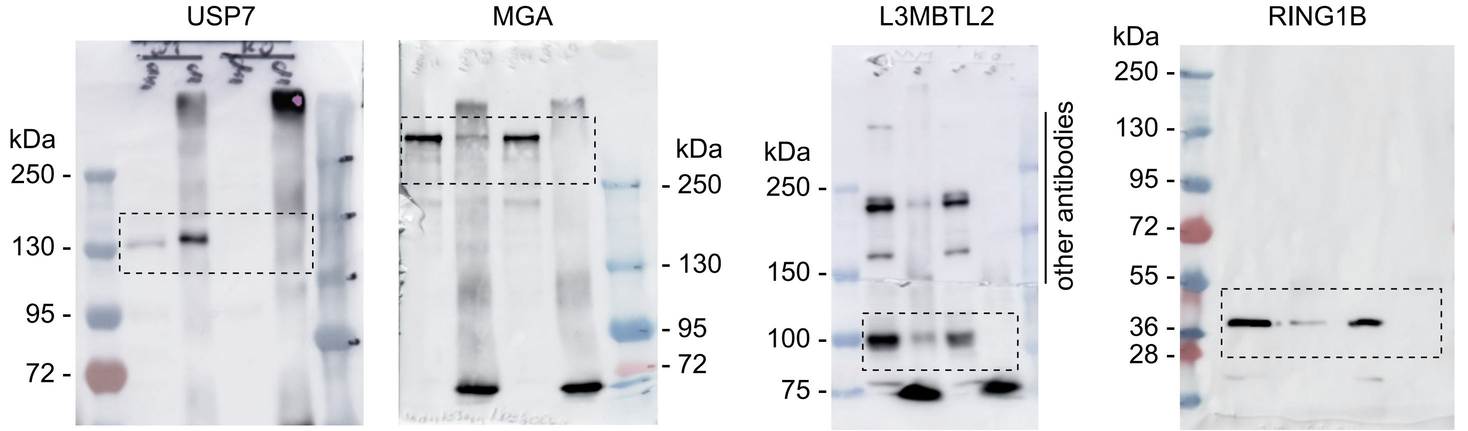

Source data_uncropped Western blots for Figure 4e

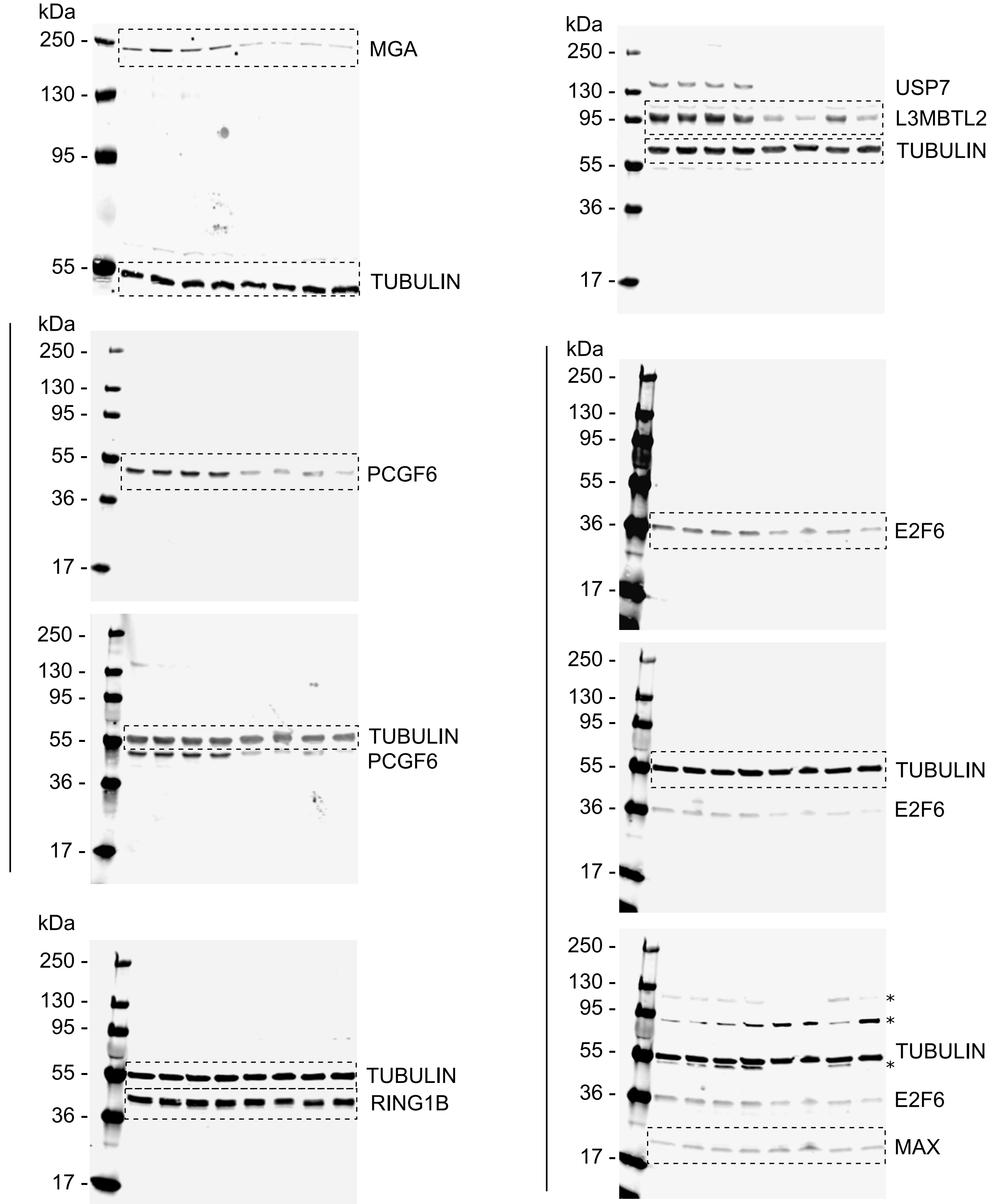

Source data_uncropped Western blots for Figure 6b

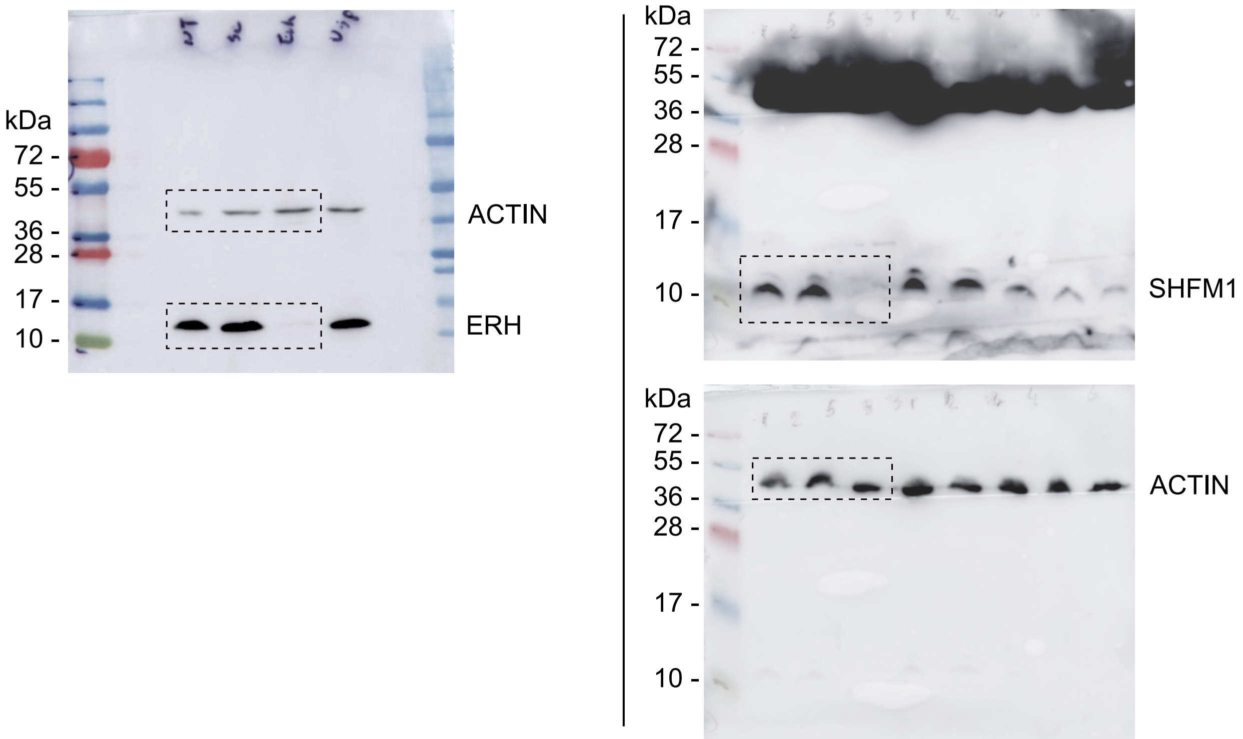

Source data_uncropped Western blots for Supplementary Figure S5c

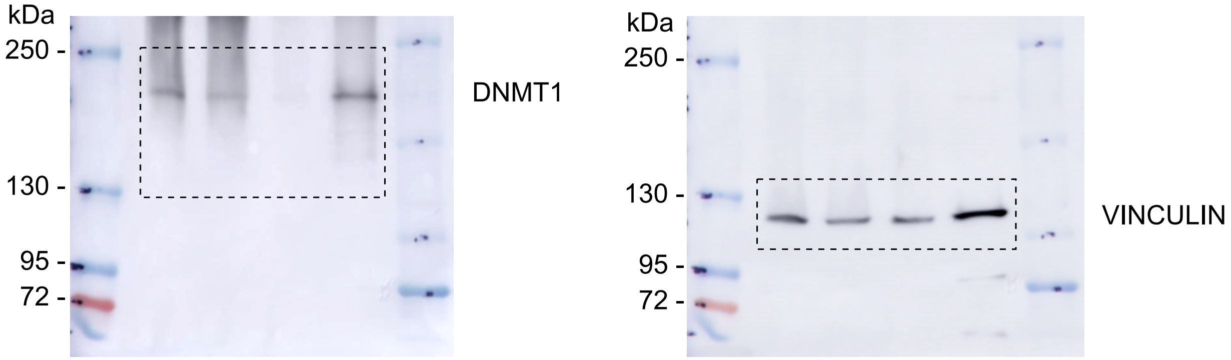

Source data_uncropped Western blots for Supplementary Figure S7b

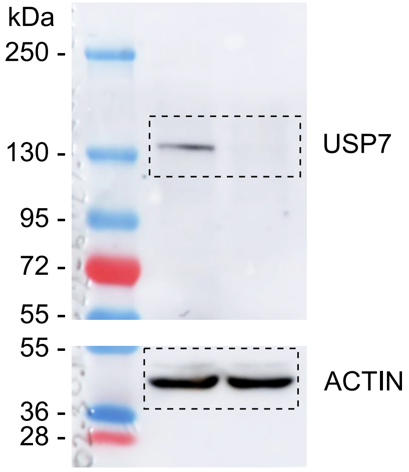

Supplement: gkad071_Supplemental_Files [file gkad071_supplemental_files.zip › Supplementary Figures.pdf]
